# A Randomized Placebo-Controlled Phase Ia Malaria Vaccine Trial of Two Virosome-Formulated Synthetic Peptides in Healthy Adult Volunteers

**DOI:** 10.1371/journal.pone.0001018

**Published:** 2007-10-10

**Authors:** Blaise Genton, Gerd Pluschke, Lukas Degen, Andreas R. Kammer, Nicole Westerfeld, Shinji L. Okitsu, Sandro Schroller, Penelope Vounatsou, Markus M. Mueller, Marcel Tanner, Rinaldo Zurbriggen

**Affiliations:** 1 Swiss Tropical Institute, Basel, Switzerland; 2 Clinical Research Center, University Hospital, Basel, Switzerland; 3 Pevion Biotech Ltd., Bern, Switzerland; London School of Hygiene & Tropical Medicine, United Kingdom

## Abstract

**Background and Objectives:**

Influenza virosomes represent an innovative human-compatible antigen delivery system that has already proven its suitability for subunit vaccine design. The aim of the study was to proof the concept that virosomes can also be used to elicit high titers of antibodies against synthetic peptides. The specific objective was to demonstrate the safety and immunogenicity of two virosome-formulated *P. falciparum* protein derived synthetic peptide antigens given in two different doses alone or in combination.

**Methodology/Principal Findings:**

The design was a single blind, randomized, placebo controlled, dose-escalating study involving 46 healthy Caucasian volunteers aged 18–45 years. Five groups of 8 subjects received virosomal formulations containing 10 µg or 50 µg of AMA 49-CPE, an apical membrane antigen-1 (AMA-1) derived synthetic phospatidylethanolamine (PE)-peptide conjugate or 10 ug or 50 ug of UK39, a circumsporozoite protein (CSP) derived synthetic PE-peptide conjugate or 50 ug of both antigens each. A control group of 6 subjects received unmodified virosomes. Virosomal formulations of the antigens (designated PEV301 and PEV302 for the AMA-1 and the CSP virosomal vaccine, respectively) or unmodified virosomes were injected i. m. on days 0, 60 and 180. In terms of safety, no serious or severe adverse events (AEs) related to the vaccine were observed. 11/46 study participants reported 16 vaccine related local AEs. Of these 16 events, all being pain, 4 occurred after the 1^st^, 7 after the 2^nd^ and 5 after the 3^rd^ vaccination. 6 systemic AEs probably related to the study vaccine were reported after the 1^st^ injection, 10 after the 2^nd^ and 6 after the 3^rd^. Generally, no difference in the distribution of the systemic AEs between either the doses applied (10 respectively 50 µg) or the synthetic antigen vaccines (PEV301 and PEV302) used for immunization was found. In terms of immunogenicity, both PEV301 and PEV302 elicited already after two injections a synthetic peptide-specific antibody response in all volunteers immunized with the appropriate dose. In the case of PEV301 the 50 µg antigen dose was associated with a higher mean antibody titer and seroconversion rate than the 10 µg dose. In contrast, for PEV302 mean titer and seroconversion rate were higher with the lower dose. Combined delivery of PEV301 and PEV302 did not interfere with the development of an antibody response to either of the two antigens. No relevant antibody responses against the two malaria antigens were observed in the control group receiving unmodified virosomes.

**Conclusions:**

The present study demonstrates that three immunizations with the virosomal malaria vaccine components PEV301 or/and PEV302 (containing 10 µg or 50 µg of antigen) are safe and well tolerated. At appropriate antigen doses seroconversion rates of 100% were achieved. Two injections may be sufficient for eliciting an appropriate immune response, at least in individuals with pre-existing anti-malarial immunity. These results justify further development of a final multi-stage virosomal vaccine formulation incorporating additional malaria antigens.

**Trial Registration:**

ClinicalTrials.gov NCT00400101

## Introduction

Apart from plans to develop a radiation-attenuated sporozoite vaccine [1], vaccine development against *Plasmodium falciparum* malaria is focusing largely on subunit vaccine technologies.[2] It is thought that an effective malaria subunit vaccine will have to incorporate antigens against several development stages of the parasite. A combination of immune responses against sporozoites, infected liver cells, merozoites and infected red blood cells may be required to achieve substantial protective activity .[2] Attempts to produce such a multi-stage subunit vaccine against malaria have so far met with limited success, indicating that new strategies both for the targeting of the immune response to suitable antigenic determinants of the parasite and for the safe and appropriate delivery of antigens are required. We are addressing these problems by developing synthetic peptide structures that elicit antibodies against the native conformation of the malaria antigens [3]–[5], and by displaying them as PE-peptide conjugates on the surface of immunopotentiating reconstituted influenza virosomes (IRIV) as carrier and adjuvant system. [3]; [6]

IRIVs are spherical, unilamellar vesicles, prepared by detergent removal from a mixture of natural and synthetic phospholipids and influenza surface glycoproteins. The haemagglutinin of the influenza virus is a fusion-inducing membrane glycoprotein, which facilitates antigen delivery to immunocompetent cells. IRIVs represent an universal antigen-delivery system for multi-component subunit vaccines, since antigens can be either attached to their surface to elicit CD4 T cell and antibody responses or encapsulated in their lumen to elicit CD8 T cell responses. They have an excellent safety profile and are already registered for human use, as two virosomal vaccines, the influenza vaccine Inflexal V® and the hepatitis A vaccine Epaxal® are commercialized.[7] More than 20 million doses of Epaxal or Inflexal V have been applied so far. These virosomal vaccine formulations are able to induce specific immunity without inducing a non-specific inflammatory response and have therfore an excellent local tolerability not only in adults but also in children [8]–[10].

We have previously shown that synthetic peptide vaccine antigens which bypass many of the problems associated with the production of stable recombinant protein-based formulations can be developed by iterative cycles of compound design, synthesis and immunological profiling.[3]–[6] Sequential rounds of optimization may lead to candidate antigens which elicit primarily antibodies that contribute to immune protection when displayed as phosphatidylethanolamine (PE)-peptide conjugates on the surface of IRIVs. CSP of *P. falciparum* is a leading sporozoite candidate antigen for inclusion in a malaria subunit vaccine. In a stepwise medicinal chemistry lead optimization process [3], [11]–[13], we have developed the conformationally constrained synthetic compound UK-39, which has best possible structural and antigenic similarity to the immunodominant NANP-repeat region of native CSP. PEV302, the virosomal formulation of UK-39, elicits high titers of sporozoite cross-reactive antibodies in mice and rabbits and these antibodies inhibit invasion of liver cells by *P. falciparum* sporozoites (Pluschke et al, personal communication). AMA-1 of *P. falciparum* is a leading merozoite candidate antigen. Its ectodomain can be divided into three subdomains each with disulfide bond-stabilized structures. Since the majority of antibodies raised against the ectodomain appear to recognize epitopes in domain I that are strain-specific, we have develop a synthetic antigen based on the more conserved loop-I of domain III, which interacts with the erythrocyte membrane protein Kx.[14] PEV301, the virosomal formulation of the optimized loop-I derived cyclized peptide antigen AMA49-C1, elicits high titers of blood stage parasite cross-reactive antibodies in mice and rabbits and these antibodies recognize primarily discontinuous epitopes comprising conserved sequence stretches of AMA-1.[4] Monoclonal antibodies generated from PEV301 immunized mice have shown *P. falciparum* blood stage growth inhibitory activity *in vitro*.[4]

Here we describe the first clinical profiling of virosomal formulations of two optimized synthetic antigens, the circumsporozoite protein (CSP) repeat region derived PE-peptide conjugate UK-39 and the apical membrane antigen-1 (AMA-1) derived PE-peptide conjugate AMA49-C1.[4] The specific objectives of the Phase l trial were to demonstrate the safety, tolerability and immunogenicity of PEV301 and PEV302 incorporating two different amounts of PE-peptide conjugate (10 µg or 50 µg per dose) given alone or in combination (50 µg of each conjugate).

## Methods

This prospective phase I, single blind, randomized, placebo controlled, dose-escalating study was conducted in 46 healthy adult volunteers at the Clinical Research Center, University Hospital, Basel, Switzerland.

The protocol for this trial and supporting CONSORT checklist are available as supporting information; see Checklist S1 and Protocol S1.

### Participants

Healthy volunteers of both sexes, aged between 18 and 45 years, with a BMI >18.5 and <30 were included if they gave written informed consent. Volunteers were excluded if they lived in the past in a malaria endemic area, had visited such an area in the last 12 months, had a history of clinical malaria, had used any investigational or non-registered drug or vaccine within 30 days preceding the first dose of study vaccine, had acute or chronic, clinically significant pulmonary, cardiovascular, hepatic or renal functional abnormality, as determined by physical examination or laboratory screening tests, had diabetes, had history of chronic alcohol consumption and/or intravenous drug abuse, had any confirmed or suspected acquired immunosuppressive or immunodeficient condition, including human immunodeficiency virus (HIV) infection, or history of congenital or hereditary immunodeficiency, had a history of allergic disease or reactions likely to be exacerbated by any component of the vaccine, had acute disease at the time of enrolment, were under immunosuppressive drugs within six months prior to the first vaccine dose, were under chronic drug therapy, were pregnant or planning to become pregnant for female volunteers.

### Vaccine

Two virosome-formulated *P. falciparum* malaria vaccines PEV301 (incorporating the AMA-1 derived PE-peptide conjugate AMA49-C1) and PEV302 (incorporating the CSP derived PE-peptide conjugate UK-39) were produced according to the rules of GMP. Tests for sterility, pyrogenicity, immunogenicity in animals, stability and chemical composition were performed on the vaccine lots used in this trial. Each dose was composed of 10 or 50 µg AMA49-C1 or UK-39, 10 µg *Influenza* haemaglutinine, 100 µg phospholipids and PBS ad 0.500 ml.

During the course of the study, stability tests were performed. We identified an unusual curve profile of PEV301 analyzed by HPLC. Validated or standardized potency tests were not available at that stage, but immunization studies in mice have shown that the clinical lot induced the expected humoral immune response. Nevertheless, the potential stability problem may have slightly impacted on the immunogenicity of this component, but has been solved since then, thanks to lyophilization of the vaccine candidate.

### Intervention

Five groups of 8 participants received virosomal formulations containing 10 ug or 50 ug of AMA49-C1, an AMA-1 derived synthetic phosphatidylethanolamine (PE)-peptide conjugate, or 10 ug or 50 ug of UK-39, a CSP derived synthetic PE-peptide conjugate, or 50 ug of both antigens each. Vaccine formulations contained different amounts of PE-peptide conjugate, but throughout the same amounts of IRIV per dose. The combination of PEV301 and PEV302 contained the double amount of influenza protein. A control group of 6 participants received unmodified virosomes. Virosomal formulations of the antigens or unmodified virosomes. The trial vaccines and comparator were administered i.m. in the left arm on day 0, in the right one on day 60 (±5 day), and the left one on day 180 (±5 days).

### Objectives

The aim of the study was to prove the concept that virosomes can be used to elicit high titers of antibodies against synthetic peptides derived from malaria antigens. The objectives were to demonstrate the safety and immunogenicity of two virosome-formulated *P. falciparum* protein derived synthetic peptide antigens given in two different doses alone or in combination. The trial was designed to investigate whether there was a dose effect of the antigens, and whether the combination of the two antigens led to beneficial, neutral or deleterious effects on safety and immunogenicity.

### Outcomes

#### Safety

Occurrence of local and systemic adverse events assessed by the physician in charge for 30 minutes, and on days 2 (or 3) and 7 (±2 days) after each vaccination, and by the subject him/herself on a diary card for 4 days. Intensity of local adverse events was graded as follows: for pain, 0 = absent, 1 = painful on touch, 2 = painful when moved, 3 = spontaneously painful; for redness and swelling, 0 = ≤5, 1 = >5-≤20 mm, 2 = >20-≤50 mm, 3 = >50 mm. For all other adverse events, grading and reporting was done according to Common Terminology Criteria for Adverse Events v3.0 (CTCAE). Haematological and biochemical analysis was carried out at screening visit (baseline), 7 (±2) and 21 (±5) days after each vaccination.

#### Immunogenicity

Antibody titer measured by ELISA against AMA49-C1 and UK-39 during the screening visit and on days 21 (±5 days), 60 (±3) (2^nd^ vaccination), 81 (+5), 180 (+3) (3^rd^ vaccination) and 201 (+5), as well as one year after the third vaccination (the latter for groups PEV302 10 µg, PEV301 50 µg (n = 8; group C) and PEV301 50 µg+PEV302 50 µg.

#### Sample Size

The sample size of this pilot study was determined by the requirement to prove the principle that the virosome-formulated synthetic peptides were safe and immunogenic. An overall sample size of 40 is generally considered appropriate in first Phase I trials to estimate the incidence rate of frequent AEs with an acceptable accuracy, allowing for dropouts. The study was not powered to ensure that differences in safety or immunogenicity between regimens would be statistically significant.

### Randomization–sequence generation

Eligible study participants were randomly allocated in six groups: PEV301 10 µg (n = 8; group A), PEV302 10 µg (n = 8; group B), PEV301 50 µg (n = 8; group C), PEV302 50 µg (n = 8; group D), PEV301 50 µg+PEV302 50 µg (n = 8; group E) or unmodified virosomes (IRIVs) serving as controls (n = 6; group F). Sequence generation for the randomization was computer performed (SAS software). The first 18 volunteers were stratified into males and females and then randomized by two blocks of 9 (1 block = females, 1 block = males) in 3 groups: PEV301 10 µg (n = 8), PEV302 10 µg (n = 8), and IRIVs alone (control; n = 2). Five weeks later, another 18 volunteers followed the same procedure: PEV301 50 µg (n = 8), PEV302 50 µg (n = 8), and IRIVs (n = 2). Five weeks after the 2^nd^ vaccination of previous groups, 10 volunteers were randomized in blocks of 5 to either the combination of PEV301 & PEV302 50 µg (n = 8) or IRIVs (n = 2).

### Randomization–Allocation concealment

Allocation concealment was done using sealed envelopes with numbers (1 to 46) corresponding to the sequence of assignment to the study. All procedures for screening and group allocation of one subject were completed before the next subject was seen.

### Randomization–Implementation

The randomization was done by PV, statistician at the Swiss Tropical Institute, who did not have any contact with the clinicians or the participants. The physician in charge of the screening and inclusion of the participants opened the envelopes sequentially, using one block for each sex.

### Blinding

The study was single blind (participant unaware of product applied).

### Laboratory methods

ELISA polysorp microtiter plates (Nunc, Dr. Grogg, Stetten-Deiswill, Switzerland) were coated at 4°C overnight with 10 µg/ml AMA49-C1 (for PEV301) or UK-39 (for PEV302) in PBS, pH 7.2. Wells were then blocked with 5% milk powder in PBS for 2 h at 37°C followed by three washings with PBS containing 0.05% Tween-20. Plates were then incubated with two-fold serial dilutions of human serum starting with 1∶50 in PBS containing 0.05% Tween-20 and 0.5% milk powder for 2 h at 37°C. After washing, the plates were incubated with horseradish-peroxidase-conjugated goat anti-human IgG antibodies (KPL, Socochim, Lausanne, Switzerland) (1∶2000 in PBS containing 0.05% Tween-20) for 1 h at 37°C and then washed. Citrat-buffer containing 4 mg/ml 1,2-diaminobezene substrate (OPD; Fluka, Sigma, Buchs, Switzerland) and 0.01% H_2_O_2_ was added and incubated at room temperature. After 10 minutes the reaction was stopped by addition of sulphuric acid (Merck, Darmstadt, Germany) to reach a final concentration of 0.5M. The optical density (OD) of the reaction product was recorded at 492 nm using a microplate reader (SpectraMax plus, Bucher Biotech, Basel, Switzerland). Titration curves were registered using Softmax PRO software. Endpoint titers were calculated by comparing the ELISA OD of the test serum with the ELISA OD of a negative serum pool. Endpoint titer is last serum dilution where the OD_test sera_≥2×OD_negativ serum_.

### Statistical Methods

The *safety analysis population* included all participants who received at least one injection, independently from protocol deviations. Data collected from drop-outs were used for the statistical analysis until the date of discontinuation. Safety of the injected study materials was determined as the incidence of adverse events and the occurrence of significant clinical, haematological and biochemical abnormalities during the procedure and at the intervals indicated in the schedule of assessments. The safety analysis population listings were made of the safety data collected at each time point.

The *Per Protocol (PP) immunogenicity analysis population* included all participants, who received the three doses of the allocated product in the allowed intervals and timely attended all the scheduled blood sampling visits. The *Intention-to-treat (ITT) immunogenicity analysis population* included all participants and all time points, independently from protocol deviations (which mainly consisted in a 1–2 day deviation from the allowed time interval). Data collected from drop-outs were included for *ITT* analysis until the date of discontinuation.

The criteria for the evaluation of immunogenicity of PEV301 and PEV302 were the results of antibody titers by Elisa against AMA49-C1 and UK-39. ELISA responders were defined as participants with a minimum titer ≥1∶500 and a >2 fold increase after immunization.

The geometric means and 95% confidence intervals of the antibody titers determined by ELISA (total IgG) for PEV 301 and PEV 302 in the four different groups were calculated separately for each time point and study group. The statistical significance of the vaccine component effects was tested using a Wilcoxon test to compare the median of the antibody titers between group A (PEV301 10 µg) and 3 (PEV301 50 µg), group A (PEV301 10 µg) and 5 (PEV301&302 50 µg) and group C (PEV301 50 µg) and 5 (PEV301&302 50 µg) at each study week assessment. Same was done for the respective groups of PEV302. The Fishers exact test was applied to compare the proportion of respondents between the groups above and at each time point with the exception weeks 9/10 and 26/27 where the titre could not be determined.

Indices of antibody response were expressed as the ratio of the antibody concentration obtained by Elisa at the 4, 12/14 and 29/30 weeks with reference the baseline measurement point. The geometric mean and 95% confidence limits were calculated and the Wilcoxon test was used to compare between groups and between the follow-up times at the 4, 12/14 and 29/30 week and the baseline. P<0.05 was considered as significant.

### Ethics and regulatory bodies

The protocol was approved by the Ethikkommission beider Basel (EKBB) and the study carried out in full compliance with the international ethical guidelines for biomedical research involving human participants and the guidelines of Good Clinical Practice. Clearance for conducting the study was also given by the Swiss Agency for Therapeutic Products (Swissmedic).

Plans were made for a Data and Safety Monitoring Board (DSMB) to be established if one of the following situations would have occurred: -one or more participants experienced a serious adverse reaction assessed as related to the vaccine by the investigator, -one or more participants experienced anaphylaxis, -two or more participants in a single dose and antigen cohort experienced a severe adverse event not explained by a diagnosis unrelated to vaccination.

Participants were financially compensated for the time lost and expenses incurred by the study requirements (transport etc.).

### Data Quality Management

The study was monitored by the Pharmaceutical Medicine Unit (PMU) of the Swiss Tropical Institute (STI) according to a monitoring plan.

## Results

### Recruitment

The study was conducted from November 2003 to October 2005. 46 Caucasian participants (half female, see Methods) were enrolled as planned.

### Participant flow

**Figure 1:**
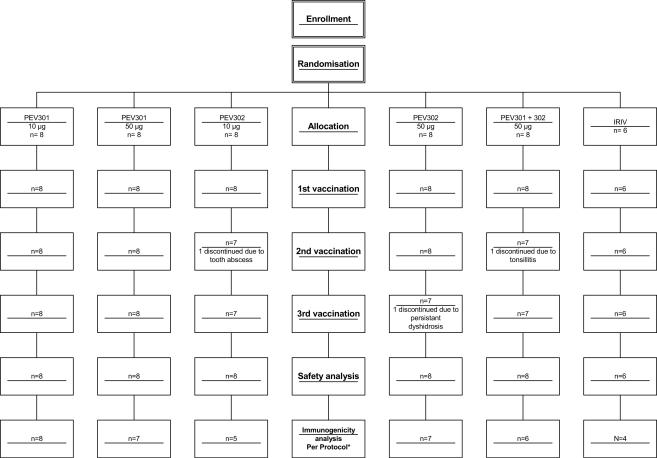
Study flowchart

All 46 participants received the 1^st^ injection, 44 the 2^nd^ and 43 the 3^rd^. Three participants discontinued participation in the trial, all of them due to a systemic AE (one of them possibly related to the study vaccine) (see below and study flowchart on figure 1).

### Baseline data

Demographic and other baseline characteristics of the population enrolled in the study are listed in Table 1.

**Table 1:** Demographic characteristics at baseline.

	PEV301		PEV302		PEV301+302	IRIV
	10 µg	50 µg	10 µg	50 µg	50 µg of each	
	N = 8	N = 8	N = 8	N = 8	N = 8	N = 6
**Age** (years)						
Mean	26	27	28	28	25	26
Min-Max	22–37	22.–40	21–42	20–40	20–30	20–42
**Weight** (kg)						
Mean	68.1	68.7	71.3	69.4	71.8	70.1
Min-Max	50.9–82.5	60.8–84.0	56.5–106.3	49.7–96.4	55.0–86.5	57.0–80.6
**BMI**						
Mean	22.6	22.9	23	22	23.8	23.7
Min-Max	19.6–25.5	20.4–28.7	19.3–29.8	19.2–26.2	20.4–29.8	20.0–27.4

### Numbers analyzed

All 46 participants were included in the safety analysis population after 1^st^ injection, 44/46 after 2^nd^ injection and 43/46 after 3^rd^ injection. 37/46 participants constituted the per-protocol immunogenicity analysis population (see study flowchart on figure 1 for details). Protocol deviations leading to exclusion from the latter population were 5 times follow-up visit dates that slightly differed from the planned schedule and once the 2^nd^ vaccination that was slightly delayed (1 subject in PEV301 50 µg , 2 in PEV302 10 µg , 1 in PEV301 & 302 50 µg and 2 in IRIV).

### Outcomes and estimation

#### Safety

No serious or severe AE occurred after the 1^st^, 2^nd^ or 3^rd^ vaccination. Exclusively pain was reported as local AE, i.e. no subject experienced at any time redness and/or swelling at the injection site. In total, 16 local AEs were reported by 11 study participants (3 from PEV301 10 µg , 2 from PEV301 50 µg , 2 from PEV302 50 µg , 2 from PEV301 & 302 50 µg and 2 from IRIV) during the period of the trial. Of these 16 events, 4 occurred after the 1^st^, 7 after the 2^nd^ and 5 after the 3^rd^ vaccination. No volunteer from PEV302 10 µg suffered from local AEs. Ten events (62.5%) were of mild and 6 (37.5%) of moderate intensity. All listed local vaccine related AEs completely resolved without sequelae within maximal 3 days after injection and no intervention was required to treat any of the local AEs.

In total 69 systemic AEs (6 possibly related and 63 either unrelated, or unlikely related to the study vaccine) were reported by 31 participants after the 1^st^ vaccination, 44 systemic AEs (10 possibly related and 34 unrelated/unlikely related) by 22 participants after the 2^nd^ immunization, and 18 systemic AEs (6 possibly related and 12 unrelated/unlikely related) by 13 participants after the 3^rd^ injection. One serious, vaccine unrelated, adverse event was reported in the PEV302 10 µg group after the 1^st^ immunization (hospitalization for tooth abscess). This subject and two others voluntarily discontinued participation in the trial, the first two prior to the 2^nd^ and the last prior to the 3^rd^ vaccination. Only one was graded as possibly related by the investigator [described as dishydrosis (dryness of the skin), which first appeared with mild intensity on the right palm and developed thereafter to moderate intensity on both hands and feet].

The type and distribution of vaccine related systemic AEs is shown in Table 2. Generally, no difference in the distribution of the systemic AEs between either the doses applied (10 respectively 50 µg) or the synthetic antigens (PEV301 and PEV302) was found. No possibly related systemic AE was experienced by volunteers from the IRIV group and less flu-like symptoms were reported in the IRIV group when compared to the verum groups.

**Table 2:** Vaccine-related systemic adverse events

Vaccine	PEV301					PEV302					PEV301 & 302		IRIV	
	10 µg		50 µg			10 µg			50 µg		50 µg each			
Stdy group	n = 8		n = 8			n = 8			n = 8		n = 8		n = 6	
**After 1^st^ immunization**														
*Headache*	–			**1**	12.5%		**2**	25.0%	–		–		–	
Nasopharyngitis	**1**	12.5%		–			–		–		–		–	
Rhinitis				**1**	12.5%		–		–		–		–	
Dyshidrosis	–			–			–		**1**	12.5%	–		–	
**Number of participants with at least one AE**	1/8	12.5%		2/8	25.0%		2/8	25.0%	1/8	12.5%	0/8	0%	0/6	0%
**After 2^nd^ immunization**														
Headache	–			**1**	12.5%		–		**1**	12.5%	–		–	
Pharyngolarnyngeal pain	–			**2**	25.0%		–		–		–		–	
Ear pain	–			**1**	12.5%		–		–		–		–	
Rhinitis	–			**1**	12.5%		–		–		–		–	
Nausea	–			–			–		**1**	12.5%	–		–	
Vertigo	–			–			–		**1**	12.5%	–		–	
Fatigue	–			–			–		**1**	12.5%	–		–	
Malaise	–			–			–		**1**	12.5%	–		–	
**Number of participants with at least one AE**	0/8	0%		2/8	25.0%		0/7	0%	2/8	25.0%	0/7	0%	0/6	0%
**After 3^rd^ immunization**														
*Pyrexia*	–			**1**	14.3%		–		–		–		–	
*Rhinitis*	–			–			**1**	14.3%	–		–		–	
*Asthenia*	–			–			–		**1**	14.3%	–		–	
*Vertigo*	–			–			–		**1**	14.3%	–		–	
*Vaginal infection*	–			–			–		–		**1**	14.3%	–	
Headache	–			–			**1**	14.3%	–		–		–	
**Number of participants with at least one AE**	0/8	0.0%		1/8	12.5%		2/7	28.6%	1/7	14.3%	1/7	14.3%	0/6	0.0%

Notes: These are all solicited and unsolicited AEs reported in the CRF and in the diary cards, and judged by the clinician in charge to be possibly related to the vaccine. The AEs in *italics* are those that occurred in the 4 days following vaccination (solicited). The values in **bold** indicate the number of AEs experienced.

Only one subject was measured with a body temperature ≥38.0°C (38.0°C one day after the 3^rd^ vaccination in the PEV301 50 µg group). Most of the AEs (related or unrelated), either observed by the physician in charge, or mentioned in the diary card were of mild (39%) or moderate (59%) intensity. Only 2% experienced severe AEs. Several laboratory values were reported to be outside the normal ranges during the trial. However, none of them was judged as clinically significant by the investigator. After pooling groups and doses, the parameters most often outside the normal ranges were: red blood cells count (45 values), ALAT (29 values), CRP (19 values), white blood cells count (15 values) and haemoglobin (14 values).

#### Immunogenicity

**Figure 2:**
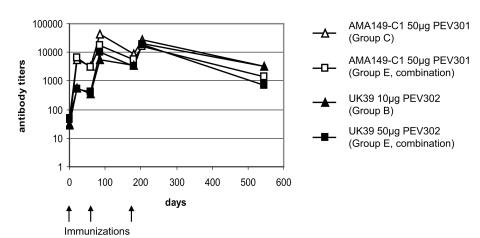
Geometric mean anti-AMA49-C1 and anti-UK-39 IgG endpoint titers in ELISA of groups immunized with optimal doses of PEV301 (50 µg) or PEV302 (10 µg), or with 50 µg each of both antigens. Sera analyzed were collected during the screening visit, at the days of the 2^nd^ and 3^rd^ vaccination, 21 days after each vaccination and one year after the third vaccination

ELISA was performed with blood samples taken during the screening visit, at the days of the 2^nd^ and 3^rd^ vaccination 21 days after each vaccination and one year after the third vaccination. Mean ELISA endpoint titers are given in Table 3 and the antibody titers of the optimal doses are shown in figure 2.

**Table 3:** Geometric means of anti-AMA49-C1 and anti-UK-39 IgG endpoint titers in ELISA and number of volunteers that seroconverted

Vaccine	PEV301		PEV302		PEV301 & PEV302		IRIV	
Dose	10 µg	50 µg	10 µg	50 µg	50 µg			
Study group	Group A	Group C	Group B	Group D	Group E		Group F*	
**Target antigen in ELISA**	AMA49-C1	AMA49-C1	UK-39	UK-39	AMA49-C1	UK-39	AMA49-C1	UK-39
**Titers after 1st injection**	77	6893	425	488	5593	392	25	77
**95% CI**	21-289	2057-28893	40-4559	10-23019	1654-18911	24-6391	25-25	
**Titers after 2nd injection**	360	45614	5658	1449	18813	11404	25	77
**95% CI**	88-1479	20170-103156	1064-30083	61-34615	6849-51676	963-135025	25-25	
**Titers after 3rd injection**	582	29117	19993	4307	16600	21945	25	77
**95% CI**	248-1367	11850-71541	2734-146208	243-76258	6391-43112	2892-166548	25-25	
**Titers 1 year after 3rd**	n.d.	3342	3200	n.d.	1213	696	n.d.	n.d.
**Nb. responders after 1st**	2/8	6/8	4/7	3/7	7/7	3/7	0/6	1/6
**Nb. responders after 2nd**	5/8	8/8	7/7	5/7	7/7	6/7	0/6	1/6
**Nb. responders after 3rd**	5/8	8/8	7/7	6/7	7/7	7/7	0/6	1/6
**Nb. responders 1 year after 3rd**	n.d.	7/8	5/6	n.d.	5/5	3/5	n.d.	n.d.

* Controls included simultaneously to Group A and 3 (301) or 2 and 4 (302)

n.d.: not done

**Figure 3:**
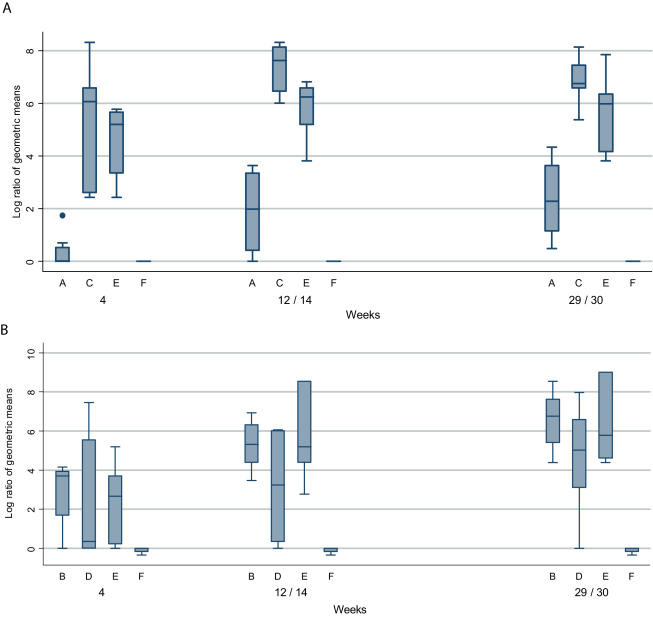
Geometric means of the ratios of geometric mean antibody endpoint titres (log scale) for samples taken three weeks after each immunization with reference to pre-vaccination titers in sera taken at the screening visit (week-1). a) anti-AMA49-C1 IgG titers (Group A = PEV301 10 µg; Group C = PEV301 50 µg; Group E = PEV301&302 50 µg, Group F = IRIV) b) anti-UK-39 IgG titers (Group B = PEV302 10 µg; Group D = PEV302 50 µg; Group E = PEV301&302 50 µg, Group F = IRIV)

Indices of responses of the two PEV301 50 µg groups were significantly higher than those of the 10 µg group at all time points (p = 0.00003 between screening visit and week 4, p = 0.00003 at week 9/10, p = 0.001 at week 26/27 and p = 0.00003 at week 29/30), demonstrating that the dose of 50 µg AMA49-C1 was superior to that of 10 µg. While all volunteers in the 50 µg groups reached a titer >1∶500 after the second and third immunization, none of the PEV301 10 µg group reached this titer (Table 3). Nevertheless, there was a significant difference between the response indices (shown in figure 3a) of PEV301 10 µg after the second and third immunization versus that of the IRIV (control group) (both p = 0.004). Differences in response indices between groups PEV301 50 µg and PEV301 & 302 50 µg were not statistically significant at weeks 4, 9/10, 12/14, 26/27 and 29/30 (p = 0.37, 0.37, 0.06, 0.07 and 0.1 respectively), indicating that the combination with PEV302 did not interfere with the response to PEV301. When comparing response indices after the first to the ones after the second immunization in the PEV301 50 µg groups, the difference was borderline significant (p = 0.09) indicating that the initial response was possibly boosted by the second administration. There was no additional benefit of having a third immunization (p = 0.7). All volunteers in the two 50 µg groups had developed an IgG response after the second immunization, whereas 62.5% (5/8) of the volunteers in the 10 µg group seroconverted. Importantly, antibody titers evaluated one year after the third immunization had remained high (Fig. 2 and Tab. 3).

With PEV302 the highest mean ELISA titers were observed after three injections, versus two for both PEV301 50 µg groups (Table 3 and figure 2). In contrast to the results with PEV301, mean ELISA endpoint titers of the group receiving 10 µg of the antigen were higher than those receiving 50 µg. Furthermore, all volunteers in the PEV302 10 µg group, but only 11/14 in the two PEV302 50 µg groups had seroconverted after two injections. After the third immunization, all volunteers in the PEV302 10 µg group, but only 11/14 in the two PEV302 50 µg groups reached an ELISA titer >1∶500. The percentage of volunteers reaching this titer increased with each immunization (Table 3). Response indices of the three groups receiving PEV302 were not significantly different (see figure 3b) [comparing group B versus E (combination) p = 0.79 between screening visit and week 4, p = 0.84 at week 9/10, p = 0.95 at week 12/14, p = 0.79 at week 26/27 and p = 0.88 at week 29/30, and comparing group D versus E (combination) p = 0.92 between screening visit and week 4, p = 0.60 at week 9/10, p = 0.34 at week 12/14, p = 0.42 at week 26/27 and p = 0.28 at week 29/30), demonstrating that the combination of PEV302 with PEV301 did not interfere with the response to PEV302. However, the index of response obtained in the PEV301 & 302 50 µg group was higher than that of PEV302 50 µg group at all time points (although not statistically significant), suggesting that the combination with PEV301 may improve the response to PEV302. When comparing response indices after the first to the ones after the second immunization in the 10 µg group, the difference was borderline significant (p = 0.056) indicating that the initial response was boosted by the second administration. There was no significant additional benefit of having a third immunization (p = 0.22). As for PEV301, antibody titers tested one year after the last immunization had remained high (Fig. 2 and Tab. 3).

In contrast to PEV301 and PEV302, no significant ELISA endpoint titers and response indices were observed against the malaria antigens in the control participants receiving IRIVs only. The results after ITT analysis are perfectly superposable to those obtained after PP analysis (data not shown).

## Discussion

### Interpretation

This is the first clinical trial that tested two new technology platforms in malaria vaccinology: synthetic peptides were optimized for eliciting parasite binding and inhibitory antibodies in iterative cycles of compound design, synthesis and immunological profiling and IRIV were tested as an immunopotentiating delivery system for PE-peptide conjugates. The results presented in this paper prove the relevance and the feasibility of the concept: i) GMP formulated material can be easily and quickly manufactured, ii) the vaccine formulation is safe, and iii) the vaccine formulation is immunogenic leading to seroconversion of all volunteers after two injections with an appropriate antigen dose.

In terms of safety, the antigen delivery platform (IRIVs) used for the tested vaccines is already commercialized and has been given to ∼20 million people, including children and infants.[15] This safety record is a major advance, since, the two malaria vaccines that have shown some efficacy in Phase IIb trials in endemic areas are formulated in adjuvants that have been given at best to only a few thousands of subjects so far.[16]–[17] Also, although probably safe, these new adjuvants are quite reactogenic.[18]–[19] As with other vaccine types, rare serious adverse events have been reported following immunizations with synthetic malaria peptides[20], but these may have occurred, because the doses of antigen were too high for the potent adjuvant used. Since the two synthetic antigens used in the present study were applied in lower doses and combined with the human approved IRIVs we expected an excellent safety profile. This was confirmed by our results that showed that the virosome-formulated PE-peptide conjugates AMA49-C1 and UK-39 as well as the combination of both are safe. The local reactogenicity was minimal with only mild or moderate pain, and no redness or swelling. This contrasts with results obtained in Phase I trials with recombinant malaria proteins formulated with ASO2A or Montanide ISA 720, or DNA vaccines in viral vectors.[19] There was no difference in the incidence rate and intensity of AEs between the participants given one or the other synthetic antigen, or both, and between the two doses, which proves the safety of the formulation.

In terms of immunogenicity, the ELISA results demonstrate that the formulation of synthetic peptides with virosomes elicited a response in all volunteers immunized with an appropriate dose. These results represent a proof of principle, i.e. they demonstrate that virosomes are a suitable antigen delivery system for malaria peptide antigens in humans and that immunogenetic restriction of the response does not represent a serious limitation for this approach. In the majority of volunteers immunized with PEV302, we observed cross-reactivity with the target proteins on the cell surface of *P. falciparum* sporozoites (data not shown). Also in the case of a minority of PEV301 immunized volunteers development of an AMA-1 specific staining pattern of malaria blood stage parasites was found in spite of variable background staining. These results demonstrate that the two synthetic antigens display the native structure of antigenic domains of the target antigens AMA-1 and CSP.

In the case of PEV301, the higher antigen dose (50 µg) was significantly superior to the lower dose (10 µg) with respect to mean antibody titers and seroconversion rate. For PEV302, the trend was reverse. These results may imply that additional antigens included in a multivalent vaccine will need a proper dose-finding assessment. In the case of PEV301 and PEV302 trends observed in animal immunogenicity studies and the clinical trial were comparable (Mueller et al., unpublished). Since the combined delivery of PEV301 and PEV302 did not interfere with the development of an immune response to either of the two components, it is hoped that the inclusion of additional antigens should not be detrimental to the overall immunogenicity. In addition, a persistent antibody titer was measured after one year.

### Generalizability

This trial was performed in malaria-naïve Caucasian adult participants. The safety profile of this vaccine formulation is likely to be quite similar when administered to children living in malaria endemic areas. Indeed, it has been regularly observed that the incidence of adverse events is lower in populations with pre-existing immunity rather than higher. Moreover, IRIV have already been successfully used as immunomodulators with antigens other than malaria in developing countries. In terms of immunogenicity, the 100% seroconversion rate achieved in malaria naïve volunteers already after only two injections with the appropriate dose of antigen is highly promising. Indeed, it is expected that the vaccine will boost pre-existing immunity and the net result should be higher immune responses in populations with natural exposure. The observation of very high antibody titers after vaccination in one volunteer that had some degree of preexisting malaria immunity at the start (and therefore wrongly included) is quite promising in this respect and justifies the planning of a Phase Ib trial in Africa.

### Overall evidence

Influenza virosomes represent an innovative human-compatible antigen delivery system that has already proven its suitability for vaccine design, such as for hepatitis A and influenza vaccines that are commercialized in many countries in the world (also for children). The present trial is the first to test the safety and immunogenicity of virosomes as carrier/adjuvant system for synthetic peptides of *Plasmodium falciparum* in humans. The excellent safety profile is in line with that observed with other virosome-formulated commercialized vaccines, and should argue for an excellent acceptability by the parents. The magnitude of the antibody responses obtained in the present trial confirm previous data from animal studies with virosome-formulated malarial antigens [21], and from human studies with antigens other than malaria. The observation that antibody responses were sustained one year after vaccination, and their avidity increased over the course of immunization (unpublished results) demonstrates the appropriateness of this technology platform. These findings justify thus the ongoing efforts to develop a virosome-formulated multi-valent multi-stage malaria vaccine. Our approach of developing optimized IRIV-formulated synthetic peptide vaccines should be generally applicable and amenable for other infectious and non-infectious diseases.

## Supporting Information

Checklist S1 CONSORT Checklist
(0.04 MB DOC)

Protocol S1 Trial Protocol
(0.33 MB PDF)
