# Supplementary material for: A Randomized Placebo-Controlled Phase Ia Malaria Vaccine Trial of Two Virosome-Formulated Synthetic Peptides in Healthy Adult Volunteers
Source: PLoS One. 2007 Oct 10;2(10):e1018. doi: 10.1371/journal.pone.0001018 (PMC2001290; doi:10.1371/journal.pone.0001018)
Supplement: Protocol S1 — Trial Protocol (0.33 MB PDF) [file pone.0001018.s002.pdf]

Confidential

CLINICAL TRIAL

Protocol No. PEV 001
Final version 1, 28.08.2003

A phase I single-blind randomized placebo controlled dose escalating study of two virosome formulated anti-malaria vaccine components (PEV 301 and PEV 302) administered alone and in combination to healthy adult volunteers

Pevion Biotech Ltd.
Bern

Confidentiality Statement

This document contains information, which is the property of Pevion Biotech Ltd., Switzerland and therefore is provided to you in confidence for review by you, your staff, an applicable ethics committee / institutional review board and regulatory authorities. It is understood that this information will not be disclosed to others without written approval from Pevion Biotech Ltd., except to the extent necessary to obtain informed consent from those persons (or their legal guardian) to whom the vaccine may be administered.

PROTOCOL NUMBER:	PEV 001, Final version 1
DATE OF PROTOCOL:	28.08.2003
PROTOCOL TITLE:	A phase I single-blind randomized placebo controlled dose escalating study of two virosome formulated anti-malaria vaccine components (PEV 301 and PEV 302) administered alone and in combination to healthy adult volunteers
SPONSOR	Pevion Biotech Ltd., Berne

RESPONSIBLE STUDY PERSONNEL:

PRINCIPAL INVESTIGATOR AND SCIENTIFIC ADVISOR
Department of Public Health & Epidemiology, Swiss Tropical Institute

Name: Dr. Blaise Genton
Address: Swiss Tropical Institute, Socinstrasse 57, CH 4002 Basel
Phone: +41-61-284 8130
Fax: +41-61-271 8654
Mail blaise.genton@hospvd.ch

CLINICAL INVESTIGATOR
Clinical Research Center Kantonsspital Basel

Name: PD Dr. Lukas Degen
Address: Clinical Research Center, Kantonsspital Basel, Petersgraben 4, CH-4031 Basel
Phone: +41 61 265 51 74
Fax: +41 61 265 53 52
e-mail lukas.degen@unibas.ch

SPONSOR and IMMUNOLOGY LABORATORY, PEVION BIOTECH LTD.
Name: Dr. Rinaldo Zurbriggen
Address: Pevion Biotech Ltd., Rehhagstrasse 79, CH-3018 Berne
Phone: +41-31-980-6417
Fax: +41-31-980-6618
e-mail rinaldo.zurbriggen@pevion.com

SCIENTIFIC ADVISOR IMMUNOLOGY, IMMUNOLOGY LABORATORY
Department of Medical Parasitology & Infection Biology, Swiss Tropical Institute

Name: Prof. Dr. Gerd Pluschke
Address: Swiss Tropical Institute, Socinstrasse 57, CH 4002 Basel
Phone: +41-61-284 8235
Fax: +41-61-271 8654
Mail gerd.pluschke@unibas.ch

STATISTICIAN
Department of Public Health & Epidemiology, Swiss Tropical Institute

Name: Dr. Penelope Vounatsou
Address: Swiss Tropical Institute, Socinstrasse 57, CH 4002 Basel
Phone: +41-61-284 8109
Fax: +41-61-271 7951
e-mail penelope.vounatsou@unibas.ch

CLINICAL RESEARCH ORGANISATION
Swiss Centre for International Health, Swiss Tropical Institute

Head of PMU at SCIH
Name: Dr. Christian Burri
Address: SCIH, Swiss Tropical Institute, Socinstrasse 57, CH 4002 Basel
Phone: +41-61-225 2661
Fax: +41-61-225 2678
e-mail christian.burri@unibas.ch

Author of Protocol and Study coordinator:
Name: Marie-Louise Mittelholzer
Address: SCIH, Swiss Tropical Institute, Socinstrasse 57, CH 4002 Basel
Phone: +41-61-225 2665
Fax: +41-61-225 2678
e-mail m-l.mittelholzer@unibas.ch

Monitor / GCP officer at SCIH:
Name: Dr. Gabriele Pohlig
Address: SCIH, Swiss Tropical Institute, Socinstrasse 57, CH 4002 Basel
Phone: +41-61-225 2662
Fax: +41-61-225 2678
e-mail gabriele.pohlig@unibas.ch

DATA MANAGEMENT
Hesperion Ltd.

Name: Dr. Milan Matejcek
Address: Hesperion Ltd., Gewerbestrasse 12/24, CH-4123 Allschwil
Phone: +41-61-487-1616
Fax: +41-61-487-1666
e-mail milan.matejcek@hesperion.com

Signature Page

PRINCIPAL INVESTIGATOR

Name:<br>Address:	Blaise Genton, PD Dr. Med., Dr. Phil nat, DTM&H<br>Swiss Tropical Institute, Socinstrasse 57<br>CH 4002 Basel<br>Switzerland
Phone:<br>Fax:	+41 61 284 28 73<br>+41 61 271 79 51

I, the undersigned, have reviewed this Protocol, including Appendices and I will conduct the clinical study as described and will adhere to GCP/ICH and all the ethical and regulatory considerations stated.

Date/Place____________________________________Signature______________________________

CLINICAL INVESTIGATOR:

Name:<br>Address:	Lukas Degen, PD Dr. Med<br>Clinical Research Center, Kantonsspital Basel, Petersgraben 4<br>CH-4031 Basel<br>Switzerland
Phone:<br>Fax:	+41 61 265 51 74<br>+41 61 265 53 52

I, the undersigned, have reviewed this Protocol, including Appendices and I will conduct the clinical study as described and will adhere to GCP/ICH and all the ethical and regulatory considerations stated. I have read and understood the contents of the Investigator Brochure.

Date/Place____________________________________ Signature______________________________

SPONSOR:

Name:<br>Address:	Rinaldo Zurbriggen, Dr. Phil nat.<br>Pevion Biotech Ltd., Rehhagstrasse 79<br>CH-3018 Bern<br>Switzerland
Phone:<br>Fax:	+41 31 980 64 17<br>+41 31 980 66 18

I have reviewed the protocol and confirm that the protocol follows the current GCP guidelines.

Date/Place____________________________________ Signature______________________________

STATISTICIAN:

Name:<br>Address:	Penelope Vounatsou, Dr. Phil. nat<br>Swiss Tropical Institute, Socinstrasse 57<br>CH 4002 Basel<br>Switzerland
Phone:<br>Fax:	+41 61 284 81 09<br>+41 61 271 79 51

I have reviewed the protocol and confirm that the protocol follows the current GCP guidelines.

Date/Place____________________________________ Signature______________________________

List of Abbreviations

AE(s)	Adverse Event(s)
ALAT	Alanine Aminotransferase
ASAT	Aspartate Aminotransferase
CNS	Central nervous system
CRC	Clinical research center
CRF	Case Report Form
CRP	C-reactive Protein
EKBB	Ethikkommission beider Basel
ELISA	Enzyme-linked Immunosorbent Assay
ELISPOT	Enzyme-linked Immunospot Assay
EMEA	European Medicines Evaluation Agency
ENT	Ear Nose Throat
GCP	Good Clinical Practice
HIV	Human immunodeficiency Virus
IB	Investigator's Brochure
ICH	International Conference on Harmonisation
IFA	Immunofluorescence Assay
IgG	Immunoglobulin G
IRB/IEC	Institutional Review Board/Independent Ethics Committee
IRIV	Immunopotentiating Reconstituted Influenza Virosomes
PMU	Pharmaceutical Medicine Unit
SAE(s)	Serious Adverse Event(s)
SOP	Standard Operating Procedure
STI	Swiss Tropical Institute

Synopsis

**STUDY NUMBER**	**PEV 001**
TITLE OF THE STUDY	A phase I single-blind randomized placebo controlled dose escalating study of two virosome formulated anti-malaria vaccine components (PEV 301 and PEV 302) administered alone and in combination to healthy adult volunteers
CLINICAL INVESTIGATOR + STUDY CENTER	PD Dr. Lukas Degen<br>Clinical Research Center, Kantonsspital Basel<br>Petersgraben 4<br>Ch 4031 Basel<br>Switzerland
PLANNED STUDY PERIOD + CLINICAL PHASE	October 2003 to October 2004<br><br>Phase I
INDICATION AND RATIONALE	No vaccine exists today against malaria. Virosomes represent an innovative antigen delivery system, which has already proven its suitability to elicit protective immune responses against subunit vaccine components in humans. The aim of the study is to proof the concept that virosomes are a suitable delivery system for peptidomimetics. This will be investigated with two prototype synthetic *P. falciparum* malaria vaccine components. More than 5 Million virosome based vaccine units have been applied so far, proving that virosomes induce a fast and very specific immune response and are very well tolerated. Subjects will not be challenged with malaria parasites.
OBJECTIVES	Primary<br>To demonstrate the safety and tolerability of two virosome formulated malaria peptidomimetics given alone and in combination.<br><br>Secondary<br>To determine the immune response (humoral and cell mediated) against two virosome formulated malaria peptidomimetics given alone and in combination.
ENDPOINTS	Safety and Tolerability:<br>• Occurrence of local and systemic adverse events<br>• Occurrence of clinically significant hematological and biochemical abnormalities<br><br>Immunogenicity:<br>• ELISA for antibody titers against PEV 301 and PEV 302, performed by Pevion Biotech Ltd.<br>• Western Blotting and IFA for antibody titers crossreactive with *P. falciparum* parasites (blood stages and sporozoites, respectively), performed by STI<br><br>Ancillary:<br>• T-cell proliferation assays, performed at STI<br>• Parasite growth/invasion inhibition assays, performed at STI<br>• IgG isotyping, performed at STI<br>• ELISPOT and clonal T-cell analyses, performed at STI<br>• Influenza ELISA/HIT, performed at Pevion Biotech Ltd.

**METHODOLOGY**		
DESIGN		Single centre, randomized, placebo controlled, comparative, single-blind, parallel groups
SUBJECTS	NUMBER	A total number of 46 volunteers (if possible 23 males, 23 females) will be enrolled into the study in order to reach a minimum of 34 subjects evaluable for immunogenicity analysis
	POPULATION	Adult healthy volunteers
	INCLUSION CRITERIA	1. Volunteers of both sexes, aged between 18 and 45 years.<br>2. Written informed consent obtained from the volunteer.<br>3. Free of obvious health problems as established by medical history and/or clinical examination before entering the study.<br>4. Body Mass Index between 18.50 and 29.99<br>5. For female volunteers, a negative pregnancy test and an adequate contraception throughout the study duration.
	EXCLUSION CRITERIA	1. Use of any investigational or non-registered drug or vaccine within 30 days preceding the first dose of study vaccine, or planned use during the study period and safety follow-up.<br>2. Chronic administration (defined as more than 14 days) of immunosuppressants or other immune-modifying drugs

		within six months prior to the first vaccine dose. (For corticosteroids, this will mean prednisone, or equivalent, ≥ 0.5 mg/kg/day. Inhaled and topical steroids are allowed.)<br>3. Any chronic drug therapy to be continued during the study period.<br>4. Longterm residency in a malaria endemic area in the past<br>5. Visit to malaria endemic area within 12 months previous to study start<br>6. Intention to travel to malaria endemic area during the study period<br>7. History of clinical malaria<br>8. Any confirmed or suspected acquired immunosuppressive or immunodeficient condition, including human immunodeficiency virus (HIV) infection, or history of congenital or hereditary immunodeficiency.<br>9. History of allergic disease or reactions likely to be exacerbated by any component of the vaccine.<br>10. Acute disease at the time of enrolment. {Acute disease is defined as the presence of a moderate or severe illness with or without fever. All vaccines can be administered to persons with a minor illness such as diarrhea, mild upper respiratory infection with or without low-grade febrile illness, i.e., temperature <38°C (<100.4°F)}<br>11. Acute or chronic, clinically significant pulmonary, cardiovascular, hepatic or renal functional abnormality, as determined by physical examination or laboratory screening tests.<br>12. Acute or chronic diabetes<br>13. History of chronic alcohol consumption and/or intravenous drug abuse.<br>14. Pregnant or lactating female.<br>15. Female planning to become pregnant.
VACCINE group A	FORMULATION / DOSE	PEV 301, 10 µg
VACCINE group B		PEV 302, 10 µg
VACCINE group C		PEV 301, 50 µg
VACCINE group D		PEV 302, 50 µg
COMBINATION group E		PEV 301 plus 302, 50 µg of each
PLACEBO group F		Virosomes alone
VACCINATION	ROUTE OF ADMINISTRATION	i.m. in M. deltoideus, 1st left, 2nd right, 3rd left hand side
	DURATION AND FREQUENCY	A total of three injections at study days 1, 64 (57-66) and 183 (176 – 187)

CRITERIA FOR EVALUATION

SAFETY		• Occurrence of adverse events: self-assessment of local and systemic reactions for 4 days after each vaccination (diary) as well as assessment by investigator during 30 minutes after each vaccination and 2 (+1) days as well as 7 (+2) days after each vaccination.<br>• Occurrence of clinically significant hematological and biochemical abnormalities (hematological and biochemical analysis will be carried out at the screening visit (baseline), 7 (+2) and 21 (+5) days after each vaccination).<br>• Significant reduction of health status compared to baseline (as assessed by investigator at baseline, i.e. prior to 1st vaccination, during 30 minutes after each vaccination and 21 (+5) days after each vaccination.
IMMUNOGENICITY	TIMING OF SPECIMEN SAMPLING for HUMORAL RESPONSE	At baseline (during screening), at the day of the 2nd and 3rd vaccination (samples to be taken prior to vaccination), and 21 (+5) days after each vaccination.
	TIMING OF SPECIMEN SAMPLING for CELLULAR RESPONSE	At baseline (during screening), and 21 (+5) days after each vaccination. Samples have to be in the laboratory at the Swiss Tropical Institute within 4 hours.<br>Cellular response will not be assessed in groups A and B.

	EVALUATION CRITERIA	• Increase of the antibody titers both against the vaccine components and *P. falciparum* parasites
	TESTS PERFORMED	See Immunogenicity & Ancillary endpoints
PROCEDURE	Volunteers will be screened, enrolled and followed by the clinical investigator, PD Dr. Lukas Degen at the Clinical Research Centre of the Kantonsspital Basel.<br>At the **screening-visit** (days -7 to -5) and after written informed consent has been obtained, the eligible healthy subjects are randomized to **vaccination** with **either three injections** of **PEV 301** 10 µg (Group A) or **PEV 302** 10 µg (Group B) or **PEV 301** 50 µg (Group C) or **PEV 302** 50 µg (Group D) or **PEV 301** 50 µg **plus PEV 302** 50 µg (Group E) or virosomes alone (group F) at days **1(+2), 64(+2) and 183(+2).**<br>If possible 23 men and 23 women should be enrolled; however, if it is not possible to have an equal sex distribution, the groups can be filled with participants of either sex.<br>The 18 volunteers recruited in October will first be stratified into males and females and then randomized in blocks of 9 (1 block = females, 1 block = males) into 3 groups: Group A (n= 8) will be vaccinated with 10 µg PEV 301, Group B (n = 8) will be vaccinated with 10µg PEV 302, and 2 volunteers will receive virosomes alone (Group F)<br>5 weeks later again 18 volunteers have to be recruited, they first will be stratified into males and females and then randomized into 3 groups: group C (n = 8) will be vaccinated with 50 µg PEV 301, group D (n = 8) with 50 µg PEV 302, and 2 volunteers will receive virosomes alone (Group F).<br>5 weeks after the 2nd vaccination of groups C and D 10 volunteers will be randomized in blocks of 5 to either group E (n = 8) receiving the combination of 50 µg PEV 301 plus 50 µg PEV 302 or group F (n = 2) receiving virosomes alone.<br>**Immunogenicity** assessments for **humoral immune response** are made at baseline (screening blood sample on days –7 to -5), on the days of the 2nd and 3rd vaccination (pre-vaccination samples) as well as 21 (+5) days after each vaccination.<br>**Cell mediated immune response** will be assessed in groups C, D, E and F only, at baseline (during screening), and 21 (+5) days after each vaccination.<br>**Safety assessments** are made by the investigator at baseline (before the 1st immunization) and before the 2nd and 3rd immunization as well as 2 (+1) and 7 (+2) days after each immunization.<br>After each immunization the volunteers have to remain in the clinic for a 30 minutes period for the assessment of local and systemic reactions.<br>The volunteers themselves have to fill an adverse event report form (diary card) for a 4 day observation period after each vaccination. Health status will be checked before each immunization, and on days 21 (+5) 85 (+2) and 204 (+2).<br>Standard blood chemistry and hematology will be assessed at the screening visit (days –7 to –5), prior to each vaccination as well as 7 (+2) and 21 (+5) days after each vaccination.	
INTERIM REPORT	An interim analysis will be performed after obtaining the results following the second injection of vaccine. Data will be collected in study weeks 12-14 (visit 9). If no humoral or cell mediated immune response is seen or the response considered sufficient in one of the study groups after 2 vaccinations, the study for this group will be terminated, i.e. no 3rd vaccination will be given.	
STATISTICAL METHODS AND EVALUATION OF DATA	Demographic data of each study group will be tabulated.<br>Safety data: Listings will be made of the safety data collected at each time point.<br>Descriptive statistics will be used to analyze adverse events (AEs) including intercurrent illnesses. The numbers of AEs and their severity will be reported using frequency tables. With frequently occurring (10 or more) event types, effects of dose and vaccine will be tested by recording the presence/absence of the event in each patient and using logistic regression models to test for differences between groups.<br>Immunogenicity data: Immunological data for each time point will be analyzed separately.<br>(i) Descriptive statistics (minimum, maximum, median, geometric mean, arithmetic mean and quartiles) will be computed for each immunological measure and each time point, separately for each group.<br>(ii) For each volunteer, the ratio of the immunological measure to that assessed at baseline (during screening) will be computed. Descriptive statistics of these ratios (minimum, maximum, median, geometric mean, and quartiles) will be computed for each immunological measure and each time point, separately for each group.<br>(iii) Wilcoxon test will be used to compare the immunological measures between doses and between group E and groups C or D (as appropriate) for each immunological measure, each time point and each antigen.	

Schedule of Assessments

	Screening Visit	Baseline Visit 1st vaccination	Follow-up Visits										
Study weeks	-1	1	1	2	4	9/10	9/10	10/11	12-14	26/27	26/27	27/28	29/30
Study days	– 7 to -5	1	3-4	8-10	22-27	57-66[a]	59-69[b]	64-75[c]	78-96[d]	176-187[e]	178-190[b]	183-197[c]	197-213[d]
Examination	Medical history in-/exclusion criteria pregnancy test (women) written informed consent	Assessment of health status (pre-vaccination)			Assessment of health status	Assessment of health status (pre-vaccination)			Assessment of health status				Assessment of health status
**Vaccination**		**0.5 mL i.m. (left)**				**0.5 mL i.m. (right)**				**0.5 mL i.m. (left)**			
Blood Sampling Safety (hematology[1] chemistry[2])	5.5 mL heparin and 2.7 mL EDTA blood			5.5 mL heparin and 2.7 mL EDTA blood	5.5 mL heparin and 2.7 mL EDTA blood	5.5 mL heparin and 2.7 mL EDTA blood		5.5 mL heparin and 2.7 mL EDTA blood	5.5 mL heparin and 2.7 mL EDTA blood	5.5 mL heparin and 2.7 mL EDTA blood		5.5 mL heparin and 2.7 mL EDTA blood	5.5 mL heparin and 2.7 mL EDTA blood
Blood Sampling Humoral Immunology	10 mL full blood (baseline values, incl. Influenza)				10 mL full blood	10 mL full blood (pre-vaccination)			10 mL full blood	10 mL full blood (pre-vaccination			10 mL full blood
Blood Sampling T-cell response[3]	25 mL EDTA blood, to be sent to STI within 4 hours				25 mL EDTA blood, to be sent to STI within 4 hours				25 mL EDTA blood, to be sent to STI within 4 hours				25 mL EDTA blood, to be sent to STI within 4 hours
Adverse Events (Interviews and diary card)		Baseline AE rate (prior to vaccination) after vaccination 30 minutes observation distribution diary card (self-assessment days 1 – 4)	Picture of injection site, if visible reaction. Recording of AEs check of diary card	Recording of AEs Collection of diary card		Baseline AE rate (prior to vaccination) after vaccination 30 minutes observation distribution diary card (self-assessment days 61 – 64)	Picture of injection site, if visible reaction. Recording of AEs check of diary card	Recording of AEs Collection of diary card		Baseline AE rate (prior to vaccination) after vaccination 30 minutes observation distribution diary card (self-assessment days 181 – 184)	Picture of injection site, if visible reaction. Recording of AEs check of diary card	Recording of AEs Collection of diary card	

1 hematology includes: hemoglobin, hematocrite, RBC count, WBC count and differential, platelets count
2 blood chemistry includes ASAT/ALAT, alk. Phosphatase, creatinine, CRP
3 T-cells will not be assessed in groups PEV 301 10 µg and 302 10 µg

a Should be 9 weeks after the 1st vaccination
b must be 2 (+1) days after vaccination
c must be 7 (+2) days after vaccination
d Must be 21 (+5) days after vaccination
e Should be 26 weeks after the 1st vaccination

Flow Chart

Week of the year	Study week	Group A (8), B (8), F (2) PEV 301/302, 10 µg	Group C (8), D (8), F (2) PEV 301/302, 50 µg	Group E (8), F (2) PEV 301 plus 302, 50 µg
45/2003	-1	Screening		
46/2003	1	Vaccination 1, safety day 3		
47/2003	2	Safety interview and lab		
48/2003	3			
49/2003	4	Safety lab, Immunology		
50/2003	5		Screening	
51/2003	6		Vaccination 1, safety day 3	
52/2003	7		Safety interview and lab	
1/2004	8			
2/2004	9		Safety lab, Immunology	
3/2004	10	Vaccination 2, safety day 3		
4/2004	11	Safety interview and lab		
5/2004	12			
6/2004	13	Safety lab, Immunology		
7/2004	14			
8/2004	15		Vaccination 2, safety day 3	
9/2004	16	**Interim analysis**	Safety interview and lab	
10/2004	17			
11/2004	18		Safety lab, Immunology	
12/2004	19			Screening
13/2004	20			Vaccination 1, safety day 3
14/2004	21			Safety interview and lab
15/2004	22		**Interim analysis**	
16/2004	23			Safety lab, Immunology
17/2004	24			
18/2004	25			
19/2004	26			
20/2004	27	*Vaccination 3, safety day 3*		
21/2004	28	*Safety interview and lab*		
22/2004	29			Vaccination 2, safety day 3
23/2004	30	*Safety lab, Immunology*		Safety interview and lab
24/2004	31			
25/2004	32		*Vaccination 3, safety day 3*	Safety lab, Immunology
26/2004	33		*Safety interview and lab*	
27/2004	34			
28/2004	35		*Safety lab, Immunology*	
29/2004	36			**Interim analysis**

10 weeks

Week of the year	Study week	Group A (8), B (8), F (2) PEV 301/302, 10 µg	Group C (8), D (8), F (2) PEV 301/302, 50 µg	Group E (8), F (2) PEV 301 plus 302, 50 µg
39/2004	46			*Vaccination 3, safety day 3*
40/2004	47			*Safety interview and lab*
41/2004	48			
42/2004	49			*Safety lab, Immunology*

Table of Contents

1 Introduction 12
1.1 Background 12
1.2 Virosomes 12
1.3 Malaria antigens 12
1.4 Rationale 13
1.5 Benefits and Risks 14
2 Objectives 14
2.1 Primary objective 14
2.2 Secondary objective 14
3 Study Design 14
4 Study Population 15
4.1 Number of subjects 15
4.2 Inclusion criteria 15
4.3 Exclusion criteria 15
4.4 Contraindications to repeated vaccination 16
5 Conduct of study 16
5.1 General study aspects 16
5.2 Detailed description of study stages/visits 17
5.2.1 Screening of potential study participants 17
5.2.2 Vaccinations 17
5.2.3 Follow-up visits 17
5.3 Blood sampling 17
5.3.1 Safety 17
5.3.2 Immunology 18
5.4 Concomitant medication/treatment 18
5.5 Subject withdrawals / dropouts / lost to follow-up 18
6 Study Vaccine(s) and Administration 18
6.1 Origin of vaccine(s) 18
6.2 Investigational Product 19
6.3 Treatment allocation and randomization 19
6.4 Dosage and administration 20
6.5 Storage 20
6.6 Method of blinding and unblinding 20
6.7 Vaccine accountability 20
6.8 Concomitant medication/treatment 20
7 Health Economics 21
8 Adverse Events (AEs) 21
8.1 Definitions 21
8.2 Surveillance period for occurrence of adverse events 22
8.3 Recording adverse events 23
8.3.1 Solicited adverse events 23
8.3.2 Unsolicited adverse events 23
8.4 Assessment of intensity 24
8.5 Assessment of causality 24
8.6 Follow-up of adverse events and assessment of outcome 25
8.7 Reporting of adverse events 25
8.8 Reporting of serious adverse events 25

8.9 Follow-up of serious adverse events 26
8.10 Pregnancy 26
8.11 Treatment of adverse events 26

9 Study Completion and Discontinuation 27

9.1 Definitions 27
9.2 Reasons for drop-out 27
9.3 Procedures for handling subjects discontinued 27
9.4 Data and Safety Monitoring Board 27

10 Data evaluation 28

10.1 Data handling 28
10.2 Primary tests 28
10.3 Secondary tests 28
10.4 Statistical hypothesis 28
10.5 Sample size calculation 28
10.6 Statistical analysis 28
10.7 Final analysis 29
10.7.1 Safety evaluation 29
10.7.2 Immunogenicity evaluation 29
10.8 Interim analysis 29
10.9 Criteria for exclusion from analysis 30

11 Ethics and regulatory considerations 30

11.1 Independent Ethics Committee 30
11.2 Informed consent 30

12 Administrative Matters 30

12.1 Protocol and Investigators Brochure (IB) 30
12.2 Confidentiality 31
12.3 Documentation and Material Supplies 31
12.4 Financial compensation 31
12.5 Monitoring 31
12.6 Source Documents and Case Report Forms (CRFs) 31
12.7 Quality assurance audit 32
12.8 Compensation for medicine-induced injury 32
12.9 Early termination 32
12.10 Publication of data and protection of trade secrets 33
12.11 Intellectual property rights 33
12.12 Entry violations 34

13 References 34

Appendix 1: Helsinki Declaration i

Appendix 2: Subject Information Sheet iv

Appendix 3: Informed Consent Agreement vii

Appendix 4: Common Terminology Criteria viii

1 Introduction

1.1 Background

It is assumed that vaccines have prevented more infectious diseases than any other medical intervention, except sanitation. With currently up to 300 million affected people, malaria continues to be one of the major burdens on public health in many tropical countries. Because of the spread of drug-resistant parasites and the appearance of insecticide-resistant mosquitoes, the development of an effective vaccine against the most severe form of malaria caused by *Plasmodium falciparum* is an urgent priority. Increased travel to endemic regions and the rapid development of parasite strains resistant to current prophylactic drugs have further increased demand for efficacious products.

Since cultivation of malaria parasites on a sufficiently large scale has turned out to be not practical for mass vaccination, efforts in malaria vaccinology are currently focused on the development of a subunit vaccine. Although a number of vaccine candidate antigens have been identified, clinical trials with first vaccine formulations including recombinant viruses, DNA vaccines and adjuvanted recombinant malaria proteins, fusion proteins and synthetic peptides, have met only limited success. Since no vaccine against malaria or against another parasitic disease for use in humans is currently available, there is the belief that for the development of such vaccines innovative approaches and technologies are required. The proposed product and technology development project represents a highly innovative approach for subunit vaccine design.

1.2 Virosomes

There is mounting evidence that the majority of natural anti-malarial immune responses (and accordingly responses to entire recombinant malaria proteins) are useless or even counterproductive. Therefore our approach is, to focus the immune response to selected protective epitopes of key malaria antigens.

We have developed new approaches and technologies for the design of epitope-focussed vaccines [1-6]. Starting point was to investigate the possibility of associating peptide or protein antigens in a defined fashion using immunopotentiating reconstituted influenza virosomes (IRIV). These are spherical, unilammelar vesicles, prepared by detergent removal from a mixture of natural and synthetic phospholipids and influenza surface glycoproteins. They have been shown to be a highly effective means of enhancing the immune response to a variety of antigens. The haemagglutinin membrane glycoprotein of the influenza virus plays a key role in the activity of IRIV. This major antigen of influenza virus is a fusion-inducing component, which facilitates antigen delivery to immunocompetent cells. In the hepatitis A vaccine Epaxal, the first licensed vaccine in which IRIV are used as a delivery system for a non-influenza antigen, the hepatitis A antigen spontaneously binds to the IRIV. We have developed and evaluated a method to couple antigens to phosphatidylethanolamine and to integrate the phospholipid-antigen conjugates into the virosome membrane during the virosome reconstitution process. In experiments in mice, the presentation of multiple copies of the antigen on the virosome surface induced strong antibody responses.

1.3 Malaria antigens

The next step was to couple mimotopes of surface loops of malaria antigens to the surface of the virosomes. Due to their inherent flexibility, linear peptides often elicit antibodies that bind to denatured proteins, but fail to recognize the same sequences in native protein structures. This is one problem that has so far hindered the application of synthetic peptides in vaccine design. Other problems are that peptides in serum have only a limited stability against proteolysis, and that their immunogenicity when administered as conjugates in human-compatible adjuvants is often weak. We have now shown that these problems may be alleviated by using conformationally defined peptidomimetics coupled to virosomes. We have

identified and optimized conformationally restricted cyclic peptide structures that mimic surface loops of two key malaria vaccine candidate antigens; the NPNA repeat region of circumsporozoite protein (CSP) and the apical membrane antigen-1 (AMA-1).
Starting with a small cyclic peptide containing portions of the NPNA-repeat region of CSP we have identified and profiled an optimized mimotope, which very efficiently elicits antibodies which cross-react with *P. falciparum* sporozoites and inhibit sporozoite invasion of human liver cells. These results demonstrated that it is possible to start from a lead structure and design a compound with optimal immunological properties in a stepwise process. With AMA-1, we used two alternative approaches to identify a lead structure for mimotope development. The first approach, immunological studies with a library of synthetic template-bound cyclic 12-mer peptidomimetics, turned out to be less successful than a second strategy in which a larger synthetic peptide structure was used as a starting point. Some of the monoclonal antibodies (mAbs) generated against this structure had growth-inhibitory activity against blood stage parasites *in vitro*.
We compared a mimotope-virosome formulation with the same mimotope presented as a multiple antigenic peptide (MAP) construct, in which several copies of the mimotope are adsorbed to an alum-adjuvant. Both formulations elicited comparable levels of anti-mimotope antibody responses in mice. However, only the antibodies against the virosome formulation bound to the parasites. This indicated that phosphatidylethanolamine-coupled antigens were located on the surface of the virosomes without their conformation being disturbed, whereas adsorption to alum dramatically disturbed their conformation.
Our joint preclinical research has identified the two virosome products PEV 301 (incorporating an Apical Membrane Antigen-1 mimotope) and PEV 302 (incorporating a Circumsporozoite Protein mimotope), which we regard as suitable components of a virosome malaria vaccine.

1.4 Rationale

No vaccine exists today against malaria. Attempts to produce a multi-stage subunit vaccine against the malaria parasite *Plasmodium falciparum* have so far met with limited success. Cumulated experience with the clinical profiling of previous malaria vaccine candidates indicates that new strategies both for the targeting of the immune response to suitable antigenic determinants of the parasite and for the safe and appropriate delivery of antigens are required. We therefore have developed an alternative approach for the design of a malaria vaccine, which is based on the delivery of peptidomimetics by immunostimulating influenza virosomes (IRIV's).
IRIV represent an adjuvant carrier system that is already incorporated in two vaccines registered for human use (EU and Canada). More than 5 million IRIV-based vaccine units have been applied so far, proving that the virosomes induce a fast and very specific immune response and are very well tolerated. Our preclinical research has demonstrated that it is possible to induce malaria parasite growth and invasion inhibitory antibodies by delivering synthetic peptidomimetics of crucial protein surface loops of different parasite development stages on the surface of IRIV to the immune system.
Sequential rounds of peptidomimetic optimization have lead to the definition of two candidate components (PEV 301 and PEV 302) for a virosomal multi-stage malaria vaccine. In experimental animals, these induce highly effective antibody responses against the merozoite and the sporozoite stages of the parasite, respectively. After an appropriate preclinical profiling, toxicological studies and GMP manufacturing, these two components are now ready for clinical testing.
Based on previous experience with the two registered IRIV-based vaccines and the highly defined nature of the two synthetic malaria peptidomimetics we expect that the candidate vaccine components will exhibit an excellent safety profile. Our approach to use conformationally defined, synthetic peptide mimetics combined with the validated virosome technology for antigen delivery should overcome many drawbacks associated with other approaches for the design of a malaria subunit vaccine.

1.5 Benefits and Risks

Volunteers will not benefit directly from participation in this study. However, it is hoped that the information gained from this study will contribute to the development of a safe and effective malaria vaccine. The general risks to participants in this Phase I study are associated with phlebotomy and with vaccination. The volume of blood drawn over the study should not compromise these otherwise healthy subjects. Potential risks include the following:
Local reactions: Mild tenderness, bruising, or fainting may result from venipuncture. An inflammatory reaction as manifested by redness, swelling, and/or tenderness may occur at the site of vaccine injection. All these local reactions have spontaneously resolved within weeks.
Systemic Reactions: Systemic reactions to immunization could theoretically occur, and include a flu-like illness with low-grade fever, chills and malaise. However, experience to date with other CSP vaccines suggests that if such reactions occur, they resolve in 12 days without therapy or limitation of daily activity. Serum sickness reactions due to deposition of antigen-antibody complexes, or idiosyncratic immune responses not dependent on immune complexes could theoretically develop resulting in damage to organs, such as the liver or kidney. Such immunemediated reactions have not been reported to date after malaria vaccines. Temporary ascending paralysis, the Guillain-Barré syndrome, may occur with any vaccine, although it is very rare.
Allergic Reactions and Anaphylaxis: As with any vaccine, allergic reactions are possible. A variety of synthetic and recombinant malaria peptide vaccines have induced systemic allergic responses, e.g., urticaria and anaphylaxis. The allergic reactions occurred after 2-3 vaccinations administered at intervals of 1 month or more, vaccines were formulated with Alhydrogel or QS-21 adjuvants, and high antigen concentrations (200 µg - 2000 µg) were inoculated with each dose. Reactions were occasionally associated with development of antigen-specific IgE antibody.
Adventitious Agents: Each lot of vaccine is tested for sterility and for either endotoxin content or pyrogenicity. The formulated product is released for use in humans when the release criteria are met, which suggests that the product is free of known microbial contamination that could infect humans. Nevertheless, the risk of unknown microbe is always present.

2 Objectives

2.1 Primary objective

To demonstrate the safety and tolerability of two virosome formulated malaria peptidomimetics given alone and in combination.

2.2 Secondary objective

To determine the immune response (humoral and cell mediated) against two virosome formulated malaria peptidomimetics given alone and in combination.

3 Study Design

This prospective phase I, single centre, randomized, single blind, placebo controlled, dose escalating study will be conducted in 46 healthy adult volunteers. Eligible study participants will be randomized into six groups: PEV 301 10 µg (group A, n = 8) or PEV 302 10 µg (group B, n = 8) or PEV 301 50 µg (group C, n = 8) or PEV 302 50 µg (group D n = 8) or PEV 301 50 µg plus PEV 302 50 µg (group E, n = 8) or virosomes alone (group F, n = 6).

Study participants will be vaccinated three times, i.e. at days 1, 64(+2) and 183(+2) and followed-up 3 times after each vaccination. If any of the study groups does not show any immune response 3 weeks after the 2nd vaccination, no 3rd vaccination will be given to that group, i.e. the study for that group will be terminated 3 weeks after the 2nd vaccination.

Groups C & D (PEV 301 50 µg; PEV 302 50 µg) will be started 5 weeks after the 1st vaccination of groups A, B.

Group E (PEV 301 plus PEV 302) will be started 5 weeks after the 2nd vaccination of groups C and D.

Group F will be distributed and serve as a control in all groups receiving the active product.

The total duration of the study for each participant is 31 weeks, the number of visits is 13.

4 Study Population

4.1 Number of subjects

A total of 46 healthy adults (if possible 23 females and 23 males) will be enrolled into the study in order to reach a minimum of 34 evaluable subjects.

Only subjects for whom the investigator believes the requirements of the protocol will be complied with (e.g. completion of the diary cards, return for follow-up visits) should be enrolled in the study.

4.2 Inclusion criteria

The following criteria should be checked at the time of study entry. If any does not apply at the time of study entry, the subject must not be included in the study:

1. Volunteers of both sexes, aged between 18 and 45 years.
2. Written informed consent obtained from the volunteer.
3. Free of obvious health problems as established by medical history and/or clinical examination before entering the study.
4. Body Mass Index between 18.50 and 29.99
5. For female volunteers, a negative pregnancy test and an adequate contraception throughout the study duration.

4.3 Exclusion criteria

The following criteria should be checked at the time of study entry. If any apply at the time of study entry, the subject must not be included in the study:

1. Use of any investigational or non-registered drug or vaccine within 30 days preceding the first dose of study vaccine, or planned use during the study period and safety follow-up.
2. Chronic administration (defined as more than 14 days) of immunosuppressants or other immune-modifying drugs within six months prior to the first vaccine dose. (For corticosteroids, this will mean prednisone, or equivalent, ≥ 0.5 mg/kg/day. Inhaled and topical steroids are allowed.)
3. Any chronic drug therapy to be continued during the study period.
4. Longterm residency in a malaria endemic area in the past
5. Visit to malaria endemic area within 12 months previous to study start
6. Intention to travel to malaria endemic area during the study period
7. History of clinical malaria
8. Any confirmed or suspected acquired immunosuppressive or immunodeficient condition, including human immunodeficiency virus (HIV) infection, or history of congenital or hereditary immunodeficiency.
9. History of allergic disease or reactions likely to be exacerbated by any component of the vaccine.
10. Acute disease at the time of enrolment. {Acute disease is defined as the presence of a moderate or severe illness with or without fever. All vaccines can be administered to

persons with a minor illness such as diarrhea, mild upper respiratory infection with or without low-grade febrile illness, i.e., temperature <38°C (<100.4°F)}

11. Acute or chronic, clinically significant pulmonary, cardiovascular, hepatic or renal functional abnormality, as determined by physical examination or laboratory screening tests.
12. Acute or chronic diabetes
13. History of chronic alcohol consumption and/or intravenous drug abuse.
14. Pregnant or lactating female.
15. Female planning to become pregnant.

If a subject signs the consent form but withdraws his/her consent after the 1st injection, he/she will abandon the study as of the date of withdrawal. The subject will attest the withdrawal on the consent form and the reason shall be noted at the end of the CRF. Data collected until the date of withdrawal will be used for the statistical analysis of safety.

If a subject is lost to follow-up or drops out, this fact shall be noted at the end of the CRF (definitions see 9.1). Data collected for such subjects will be used for the statistical analysis until the date of lost to follow-up or dropout.

4.4 Contraindications to repeated vaccination

The following adverse events constitute absolute contraindications to further administration of PEV 301 / PEV 302; if any of these adverse events occur during the study, the subject must not receive additional doses of vaccine but may continue other study procedures at the discretion of the investigator. The subject must be followed until resolution of the event, as with any adverse event (see Section 8):

- Anaphylactic reaction following the administration of vaccine(s).
- Any clinically relevant immunosuppressive or immunodeficient condition

The following adverse events constitute contraindications to administration of PEV 301 / PEV 302 at that point in time; if any one of these adverse events occurs at the time scheduled for vaccination, the subject may be vaccinated at a later date, within the time window specified in the protocol (see Schedule of Assessments Page 8), or withdrawn at the discretion of the investigator. The subject must be followed as with any adverse event (see Section 8).

- Acute disease at the time of vaccination. (Acute disease is defined as the presence of a moderate or severe illness with or without fever. All vaccines can be administered to persons with a minor illness such as diarrhea, mild upper respiratory infection with or without low-grade febrile illness, i.e., temperature <38°C.
- Temperature $\geq$ 38°C at the time of vaccination.

5 Conduct of study

5.1 General study aspects

The clinical part of the study will be conducted at the Clinical Research Centre of the Kantonsspital Basel. PD Dr. Lukas Degen is the Clinical Investigator.

It is the investigator's responsibility to ensure that the intervals between visits/contacts are strictly followed. These intervals during the active phase of the study determine a subject's evaluability in the as per protocol analyses.

5.2 Detailed description of study stages/visits

For information on time points of visits, vaccinations & checks refer to schedule of assessments page 8.

5.2.1 Screening of potential study participants

During the Screening Visit (days –7 to -5) the investigator has to check whether potential participants for this study meet the inclusion criteria. Volunteers will not be included if any of the exclusion criteria apply. Volunteers who have signed the Informed Consent Form will undergo complete physical examinations, including vitals signs (blood pressure, pulse, temperature) and body systems (cardiovascular, gastro-intestinal, CNS, ENT, respiratory, urogenital, dermatology). Demographic data will be collected, and a complete clinical history will be recorded, and laboratory tests (including HIV and influenza A titer) and a pregnancy test in female volunteers will be performed. In the event that a subject is tested positive for HIV, he/she will be informed and referred for counseling and treatment. Temperature will also be measured daily by the subject and recorded on the diary card provided.

If materials are provided by a centralized laboratory, it is mandatory that all clinical samples (including serum samples) be collected using exclusively those materials in the appropriate manner. The use of other materials could result in the exclusion of the subject from analysis. The investigator must ensure that his/her personnel and the laboratory(s) under his/her supervision comply with this requirement.

5.2.2 Vaccinations

Volunteers will be vaccinated on days 1, 64 (57 – 66) and 183 (176 – 187) respectively. The 3rd vaccination will not be applied, if 3 weeks after the 2nd vaccination no immune response is seen.
Prior to the vaccination the baseline AE rate has to be assessed by the investigator.

5.2.3 Follow-up visits

For timepoints see schedule of assessments, page 8
Study participants have to report to the study site 48 hours, i.e. on day 2 (+1) after each vaccination. If a visible local reaction occurs, the investigator has to take a picture of the injection site. He has to record any adverse events in the CRF. The investigator has to check whether the study participants fill in the diary card provided to them.

The second follow-up visit has to be performed 7 (+2) days after each vaccination. The investigator has to collect the diary card, to record any adverse events not recorded by the participant and to take blood samples for safety checks (see 5.3.4.)

The 3rd follow-up visit has to be made 22 (+5) days after each vaccination. The investigator has to assess the health status of the participant and to take blood samples for safety and immunology tests (see 5.3.4.)

5.3 Blood sampling

5.3.1 Safety

For timepoints see schedule of assessments, page 8.

The following blood samples have to be taken: 2.7 mL EDTA blood for the assessment of hematological parameters, 5.5 mL heparin blood for blood chemistry tests. Test tubes will be provided by the Zentrallabor of the Kantonsspital Basel; handling of the blood samples has to follow the instruction of the Zentrallabor, where the following analyses will be performed:

- Hematology:
  - Hemoglobin
  - Hematocrite
  - Red Blood Cell count
  - White Blood Cell count and differential count
  - Platelet count

- Blood chemistry:
  - ASAT
  - ALAT
  - Alkaline Phosphatase
  - Creatinine
  - CRP

5.3.2 Immunology

For timepoints see schedule of assessments, page 8.

10 mL full blood for the analysis of humoral immune responses, and 25 mL EDTA blood for the analysis of cellular immune responses. All blood samples will be processed at the Swiss Tropical Institute. The EDTA blood has to arrive there within 4 hours. ELISA will be performed at Pevion Biotech Ltd., the other assays at the Swiss Tropical Institute.

5.4 Concomitant medication/treatment

At each study visit/contact, the investigator should question the subject about any medication taken.

Any immunosuppressants or other immune-modifying drugs or treatments, any vaccine other than the study vaccine(s) and any antipyretics administered at ANY time during the period starting 30 days prior to the first dose of study vaccine(s) and ending one month (minimum 30 days) after the last dose of study vaccine(s) must be recorded in the CRF with trade name and/or generic name of the medication, medical indication, total daily dose, route of administration, start and end dates of treatment.

Any other concomitant medication administered prophylactically in anticipation of reaction to the vaccination must also be recorded in the CRF with trade name and/or generic name of the medication, total daily dose, route of administration, start and end dates of treatment and coded as `prophylactic'.

5.5 Subject withdrawals / dropouts / lost to follow-up

Withdrawals / dropouts will NOT be replaced.

For more information see chapter 9 "Study Completion and Discontinuation"

The subject should be informed of the right to abstain from participation in the study or to withdraw consent to participate at any time without reprisal.

6 Study Vaccine(s) and Administration

6.1 Origin of vaccine(s)

All candidate vaccines to be used have been developed and manufactured by Pevion Biotech Ltd.

The Quality Control Standards and Requirements for each candidate vaccine are described in separate release protocols/Certificate of Analysis and the required approvals have been obtained.

6.2 Investigational Product

The virosome-formulated malaria vaccines PEV 301, PEV 302 and vehicle are supplied in vials. The watery solution is slightly opaque.

PEV 301 0.500 mL Lot Nr.: 03PEVXX
Active substances: 10 resp. 50 µg AMA1 mimetic
Excipients: IRIV, PBS pH 7.4

PEV 302 0.500 mL Lot Nr.: 03PEVXX
Active substances: 10 resp. 50 µg CSP mimetic
Excipients: IRIV, PBS pH 7.4

PEV 301 + PEV 302 0.500 mL Lot Nr.: 03PEVXX
Active substances: 50 µg AMA1 mimetic, 50 µg CSP mimetic
Excipients: IRIV, PBS pH 7.4

IRIV 0.500 mL Lot Nr.: 03PEVXX
Active substances: none
Excipients: IRIV, PBS pH 7.4

The vaccines will be blistered and packed in boxes labeled with "Drug for investigational use only” (see example below):

PEV 001

50 µg PEV 301
Malaria vaccine
Drug for investigational use only
For i.m. injection

Lot:03PEVXX

Store at +2 – +8°C
Expiry date: MM.JJJJ
Pevion Biotech Ltd.

6.3 Treatment allocation and randomization

Randomization will be computer generated. The investigator will receive envelopes with numbers (1 to 46) corresponding to the sequence of assignment to the study. In the envelope the assignment to the study group will be determined.

The 18 volunteers recruited in October will first be stratified into males and females and then randomized in blocks of 9 (1 block = females, 1 block = males) into 3 groups: Group A (n= 8) will be vaccinated with 10 µg PEV 301, Group B (n = 8) will be vaccinated with 10µg PEV 302, and 2 volunteers will receive virosomes alone (Group F). If it is not possible to have an equal sex distribution, the blocks can be filled with participants of either sex.
5 weeks later again 18 volunteers have to be recruited, they first will be stratified into males and females and then randomized into 3 groups: group C (n = 8) will be vaccinated with 50 µg PEV 301, group D (n = 8) with 50 µg PEV 302, and 2 volunteers will receive virosomes alone (Group F).
5 weeks after the 2nd vaccination of groups C and D 10 volunteers will be randomized in blocks of 5 to either group E (n = 8) receiving the combination of 50 µg PEV 301 plus 50 µg PEV 302 or group F (n = 2) receiving virosomes alone.

Time	N	Dose	PEV 301	PEV 302	PEV 301+302	virosomes
November 2003	18	10 µg	8	8		2
December 2003	18	50 µg	8	8		2
March 2004	10	50 + 50 µg			8	2

6.4 Dosage and administration

PEV 301 and PEV 302 will be provided in vials. The vials for each group will be provided in individually labeled boxes.

0.5 mL PEV 301 and PEV 302 vaccines should be administered intramuscularly (i.m.) into the deltoid region of the upper arm. The first application is to be given to left, the second to right and the third to the left arm, respectively. The vaccine should not be injected into the blood vessels. The vaccination site should be disinfected with a skin disinfectant (e.g. 70% alcohol) prior to vaccination. The Investigator should use only the needles provided by the Sponsor.

The subjects will be observed closely for at least 30 minutes after vaccination, with appropriate medical treatment readily available in case of a rare anaphylactic reaction following the administration of vaccines. Any reactions that occur during this time must be recorded by the Investigator in the CRF.
The subjects will be instructed to contact the investigator immediately should they manifest any signs or symptoms they perceive as serious. They have to note any signs or symptoms they notice following the vaccinations, on a diary card provided to them.

6.5 Storage

It is essential that Pevion can be certain that the study vaccines retain their safety and potency for the duration of their assigned shelf life. All vaccines must be stored in a safe and locked place with no access for unauthorized personnel. They must be kept in a refrigerator (+2°C to +8°C) and must not be frozen.

6.6 Method of blinding and unblinding

NA

6.7 Vaccine accountability

The investigator has the obligation to fill in the respective forms provided. After approval from Pevion Biotech Ltd. Ltd, both used and unused vaccine vials should be sent back to Pevion Biotech Ltd. for destruction. The destruction may be performed at the study site using locally approved biosafety procedures and documentation.

6.8 Concomitant medication/treatment

At each study visit/contact, the investigator should question the subject about any medication taken.
Any concomitant medication administered prophylactically in anticipation of reaction to the vaccination must also be recorded in the CRF with trade name and/or generic name of the medication, total daily dose, route of administration, start and end dates of treatment and coded as `prophylactic'.

7 Health Economics

Not applicable

8 Adverse Events (AEs)

The recording of adverse events is an important aspect of study documentation. It is the responsibility of the investigator to document all adverse events according to the detailed guidelines set out below.

The subjects will be instructed to contact the investigator immediately should the subject manifest any signs or symptoms they perceive as serious during the period extending from performance of the first study procedure up to and including 6 months (minimum 180 days) after the last dose of study vaccine.

8.1 Definitions

Adverse event:

An adverse event is any untoward medical occurrence in a patient or clinical trial subject administered a medicinal product and which does not necessarily have to have a causal relationship with this treatment.

This includes any noxious, pathological or unintended change in anatomical, physiological or metabolic functions as indicated by physical signs, symptoms and/or laboratory detected changes occurring in any phase of the clinical study whether associated with the study vaccine, active comparator or placebo and whether or not considered vaccination related. This includes an exacerbation of pre-existing conditions or events, intercurrent illnesses, or vaccine or drug interaction. Anticipated day-to-day fluctuations of pre-existing conditions that do not represent a clinically significant exacerbation need not be considered adverse events. Discrete episodes of chronic conditions occurring during a study period should be reported as adverse events in order to assess changes in frequency or severity.

Adverse events should be documented in terms of signs and symptoms observed by the investigator or reported by the subject at each study visit. A medical diagnosis should be added.

Pre-existing conditions or signs and/or symptoms (including any which are not recognized at study entry but are recognized during the study period) present in a subject prior to the start of the study should be recorded in the medical history part of the subject's CRF.

Adverse events, which occur after informed consent is obtained, but prior to vaccination, will be documented in the medical history part of the subject's CRF.

Although not considered as an adverse event, hospitalization for either elective surgery related to a pre-existing condition, which did not increase in severity or frequency following initiation of the study, or for routine clinical procedures (including hospitalization for "social" reasons) that are not the result of an adverse event, must be recorded in the CRF. If the hospitalization arises from a pre-existing condition, or was planned prior to the first vaccination, it should be recorded in the medical history part of the CRF. If it was planned after the first vaccination, it should be recorded in the adverse event page of the CRF. In both cases, it should be recorded as `Hospitalization (Not an adverse event)', and the relationship to vaccination will be checked "No". These adverse events are not considered as SAE.

Serious adverse events

A serious adverse event is any untoward medical occurrence or effect that any dose

- results in death,

- is life threatening*,
- results in persistent or significant disability/incapacity†,
- requires in-patient hospitalization‡ or prolongation of existing hospitalization
- is a congenital anomaly/birth defect in the offspring of a study subject.
- is an important medical event that may jeopardize the patient or may require intervention to prevent one of the other outcomes listed above should be considered serious. (Examples of such treatments are intensive treatment in an emergency room or at home for allergic bronchospasm; blood dyscrasias or convulsions that do not result in hospitalization; or development of drug dependency or drug abuse.)

Although not considered as ‘serious adverse events', cancers should be reported in the same way as serious adverse events.

* Life threatening - definition: An adverse event is life threatening if the subject was at risk of death at the time of the event; it does not refer to an event, which hypothetically might have caused death if it were more severe.

† Disabling/incapacitating - definition: An adverse event is incapacitating or disabling if the event results in a substantial disruption of the subject's ability to carry out normal life functions. This definition is not intended to include experiences of relatively minor medical significance such as headache, nausea, vomiting, diarrhea, influenza, injection site reactions and accidental trauma (e.g. sprained ankle).

‡ Hospitalization: In general, hospitalization signifies that the subject has been detained (usually involving at least an overnight stay) at the hospital or emergency ward for treatment that would not have been appropriate in the physician's office or out-patient setting.

Hospitalization for either elective surgery related to a pre-existing condition, which did not increase in severity or frequency following initiation of the study or for routine clinical procedures (including hospitalization for "social" reasons) that are not the result of an adverse event need not be considered as adverse events and are therefore not serious adverse events.

¶ Routine Clinical Procedure definition: One, which is defined as a procedure, which may take place during the study period and should not interfere with the study vaccine administration or any of the ongoing protocol specific procedures.

N.B. If anything untoward is reported during an elective procedure, that occurrence must be reported as an adverse event, either `serious' or non-serious according to the usual criteria.

When in doubt as to whether ‘hospitalization’ occurred or was necessary, the adverse event should be considered serious.

8.2 Surveillance period for occurrence of adverse events

All adverse events occurring within one month (minimum 30 days) following administration of each dose of vaccine/ placebo must be recorded on the Adverse Event Form in the subject’s CRF, irrespective of severity or whether or not they are considered vaccination-related.

Additionally, all serious adverse events brought to the attention of the investigator during the period starting from the day of performance of the first study procedure for each subject and ending not earlier than six months (minimum 180 days) following administration of the last dose of study vaccine for that subject must be recorded. See 8.8 for instructions for recording and reporting of serious adverse events.

8.3 Recording adverse events

At each visit/assessment, all adverse events either observed by the investigator or one of his clinical collaborators or reported by the subject spontaneously or in response to a direct question will be evaluated by the investigator. Adverse events not previously documented in the diary card will be recorded in the Adverse Event form within the subjects CRF. The nature of each event, date and time (where appropriate) of onset, outcome, intensity and relationship to vaccination should be established. Details of any symptomatic/corrective treatment should be recorded on the appropriate page of the CRF.

8.3.1 Solicited adverse events

Solicited symptoms are adverse events reported by the subjects, which occur immediately after the injection, or any time between the injection and the 4-day follow-up period.

Local (injection site) adverse events

The following solicited adverse events have been reported in similar studies:

- Pain
- Redness
- Swelling

Solicited local adverse events are considered "vaccine-related".

General adverse events

This is the first study in men with PEV 301 and PEV 302, therefore, general solicited adverse events related to PEV 301 and PEV 302 will be determined in this study.

As a consistent method of soliciting adverse events, the subject should be asked a non-leading question such as:

"Have you felt different since receiving the vaccine or since the previous visit?"

Adverse events already documented in the CRF, i.e. at a previous assessment, and designated as ‘ongoing’ should be reviewed at subsequent visits, as necessary. If these have resolved, the documentation in the CRF should be completed. If an adverse event changes significantly in frequency or intensity during a study period, a new record of the event will be started.

8.3.2 Unsolicited adverse events

Unsolicited symptoms are adverse events, reported by the subjects, which begin after the 4 day follow-up period for solicited symptoms. Space on the diary card and CRF will be allocated for the recording of unsolicited symptoms. Should any systemic (general) signs or symptoms be reported, their relationship with the study vaccine will be assessed by the investigator and transcribed into the CRF.

8.4 Assessment of intensity

Intensity of the following local adverse events should be assessed as described:

Adverse Event	intensity grade	Parameter
Pain	0	Absent
	1	Painful on touch
	2	Painful when limb is moved
	3	Spontaneously painful
Redness	0<br>1<br>2<br>3	Absent<br>>5 mm<br>>20 mm<br>>50 mm
Swelling	0<br>1<br>2<br>3	Absent<br>>5 mm<br>>20 mm<br>>50 mm

For all other adverse events, grading and reporting has to be done according to Common Terminology Criteria for Adverse Events v3.0 (CTCAE). This document will be provided to the CRC of the Kantonsspital Basel by STI-PMU as part of the Investigator Folder.

8.5 Assessment of causality

Every effort should be made by the investigator to explain each adverse event and assess its causal relationship, if any, to administration of the study vaccine(s).

The degree of certainty with which an adverse event can be attributed to administration of the study vaccine(s) (or alternative causes, e.g. natural history of the underlying diseases, concomitant therapy, etc.) will be determined by how well the event can be understood in terms of one or more of the following:

- Reaction of similar nature having previously been observed with this type of vaccine and/or formulation.
- The event having often been reported in literature for similar types of vaccines.
- The event being temporally associated with vaccination or reproduced on re-vaccination.

All solicited reactions will be considered related to vaccination. Causality of all other adverse events should be assessed by the investigator using the following method:

In your opinion, is there a reasonable possibility that the adverse event may have been caused by the study vaccine(s)?

Related	suspicion that there is a relationship between vaccine and AE (without determining the extent of probability); there is a reasonable possibility that the vaccine contributed to the AE
Probable	AE occurs within a reasonable time after the administration of the vaccination and cannot be reasonably explained by other factors (i.e. clinical condition, environmental / toxic factors or other treatments)
Possible	AE occurs within a reasonable time after the administration of the vaccine but can also be reasonably explained by other factors (as mentioned above)
Unlikely	AE does not occur within a reasonable time after the administration of the vaccine (unusual time frame) and can also be reasonably explained by other factors (as mentioned above)

Unrelated — there is no suspicion that there is a relationship between vaccine and adverse event, there are other more likely causes and administration of the study vaccine is not suspected to have contributed to the AE

Non-serious and serious adverse events will be evaluated as two distinct types of events given their different medical nature. If an event meets the criteria to be determined "serious" (see Section 9.1 for definition of serious adverse event), it will be examined by the investigator to the extent to be able to determine ALL contributing factors applicable to each serious adverse event.

Other possible contributors include:
- Underlying disease
- Other medication
- Protocol required procedure
- Other cause (specify)

8.6 Follow-up of adverse events and assessment of outcome

Investigators should follow-up non-serious adverse events until the end of the study.

Outcome should be assessed as:
1 = Resolved
2 = Resolved with sequelae
3 = Ongoing at subject study conclusion
4 = Died
5 = Lost to follow up

8.7 Reporting of adverse events

All adverse events occurring within one month (minimum 30 days) following administration of each dose of vaccine must be recorded on the adverse event form in the subject's CRF, irrespective of severity or whether or not they are considered vaccination-related.

At each visit/assessment, all adverse events, either observed by the investigator or one of his clinical collaborators or reported by the subject spontaneously or in response to a direct question will be evaluated by the investigator. Adverse events not previously documented in the study will be recorded in the AE form within the subject's CRF. The nature of each event, date and time (where appropriate) of onset, outcome, intensity and relationship to vaccination should be established. Details of any corrective treatment should be recorded on the appropriate page of the CRF.

8.8 Reporting of serious adverse events

All Serious Adverse Events (SAEs) must be reported immediately by the investigator without filtration, whether considered to be associated with the study vaccine, active comparator or placebo and whether or not considered vaccination related. The investigator must report SAEs within one calendar day of becoming aware of the event by telephone, telefax or e-mail (if appropriate) to the Study Contact for Reporting Serious Adverse Events as described below. This initial notification should include minimal, but sufficient information to permit identification of the reporter, the subject, the study vaccine, adverse events, and date of onset. The investigator should not wait for additional information to fully document the event before notifying. The responsible monitor will confirm this first notification. The report is then to be followed by submission of a completed SAE Report Form provided by STI/PMU as soon as possible but at latest within 3 calendar days of the initial telephone / telefax or e-mail report detailing relevant aspects of the adverse events in question. All actions taken by the investigator and the outcome of the event must also be reported immediately. For

documentation of the SAE and, any actions taken, for outcome and follow-up reports the SAE Report Forms are to be used. Where applicable, hospital case records and autopsy reports should be obtained.

Investigators must report SAEs to the Ethikkommission beider Basel according to the "Richtlinien bezüglich der Meldung von Serious Adverse Events (SAE) an die EKBB" as published on the webpage of the EKBB (www.ekbb.ch).

**Study Contact for Reporting Serious Adverse Events**<br>**24/24 hour and 7/7 days availability**
Name, address: Marie-Louise Mittelholzer, Schweizerisches Tropeninstitut<br>Tel: 061 225 26 65<br>Fax: 061 225 26 78<br>**Outside office hours**<br>Tel: 061 811 42 57<br>Mobile phone: 076 593 83 19
**Back-up Study Contact for Reporting Serious Adverse Events**
Name: Dr. Peter Klein, Pevion Biotech Ltd. AG<br>Tel: 031 980 62 12<br>Fax: 031 980 66 18<br>Mobile phone: 079 332 61 44

8.9 Follow-up of serious adverse events

All serious adverse events must be documented and followed up until the event either resolved, subsided, stabilized, disappeared or is otherwise explained or the study subject is lost to follow-up, but not longer than 6 after the last vaccination. All follow-up activities have to be reported, if necessary on one or more consecutive SAE report forms in a timely manner. All fields with additional or changed information must be completed and the report form should be forwarded to the Study Contact for Reporting Serious Adverse Events as soon as possible but latest within 5 calendar days after receipt of the new information.
Clinically significant laboratory abnormalities will be followed up until they have returned to normal, or a satisfactory explanation has been provided. Reports relative to the subsequent course of an adverse event noted for any subject must be submitted to the Sponsor.

8.10 Pregnancy

Subjects who become pregnant during the study period (up to and including one month [minimum 30 days] after receiving the last vaccine dose) must not receive additional doses of vaccine but may continue other study procedures at the discretion of the investigator.

Subjects should be instructed to notify the investigator if it is determined after completion of the study that they became pregnant either during the study or within one month (minimum 30 days) after receiving the last vaccine dose.

A pregnancy should be followed to term, any premature terminations reported, and the health status of the mother and child including date of delivery and the child's gender and weight should be reported to Pevion Biotech Ltd. after delivery.

8.11 Treatment of adverse events

Treatment of any adverse event is at the sole discretion of the investigator and according to current Good Medical Practice. The applied measures should be recorded in the CRF of the subject.

9 Study Completion and Discontinuation

9.1 Definitions

Discontinuation from the study can occur under the following circumstances:

Screening errors:
From an analysis perspective, a 'screening error' is any subject who was enrolled into the study, i.e. was attributed a CRF with subject number, but was withdrawn (before or after randomization) prior to vaccination for reasons such as protocol violation (inclusion/exclusion criteria), 'force majeure' or withdrawal of consent.

Lost to follow-up:
From an analysis perspective, a 'lost to follow-up' is any subject who completed all protocol specific procedures up and including to Day 1, including vaccination, but was then lost to any further follow-up, (no safety information and no efficacy endpoint data ever became available)

Drop-outs:
Any subject for whom data is available for at least one follow-up visit but did not complete the study as foreseen in the protocol.

9.2 Reasons for drop-out

It should be specified on the End of Study page of the CRF, which of the following possible reasons were responsible for drop-out of the subject from the study:

- Adverse events
- Protocol violation (specify)
- Consent withdrawal, not due to an adverse event ,
- Migrated/moved from the study area
- Other (specify)

9.3 Procedures for handling subjects discontinued

Investigators should make an attempt to contact those subjects who do not return for scheduled visits or follow-up. Information gathered should be described on the Study Conclusion page of the CRF and on Medication/Adverse event forms.

9.4 Data and Safety Monitoring Board

A Data and Safety Monitoring Board (DSMB) will be established if one of the following situations occurs:

- One or more subjects experienced a serious adverse reaction assessed as related to the vaccine by the investigator
- One or more subjects experienced anaphylaxis
- Two or more subjects in a single dose and antigen cohort experienced a severe adverse event not explained by a diagnosis unrelated to vaccination. (For grading of severity see Appendix 4)

The DSMB will then analyze the data and decide on the continuation of the study.

10 Data evaluation

10.1 Data handling

The data collected with the CRFs will be entered into a database set up by Hesperion Ltd, Allschwil. Hesperion is responsible for data handling and record keeping.

The statistical analysis of the data and the preparation of tables and listings for interim and final reports will be performed by the project statistician, Dr. Penelope Vounatsou, Swiss Tropical Institute.

10.2 Primary tests

Safety and Tolerability

- Occurrence of local and systemic adverse events
- Occurrence of clinically significant hematological and biochemical abnormalities

Immunogenicity

- ELISA for antibody titers against PEV 301 and PEV 302, performed by Pevion Biotech Ltd.
- Western Blotting and IFA for antibody titers crossreactive with *P. falciparum* parasites (blood stages and sporozoites, respectively), performed by STI

10.3 Secondary tests

- T-cell proliferation assays, performed at STI
- Parasite growth/invasion inhibition assays, performed at STI
- IgG isotyping, performed at STI
- ELISPOT and clonal T-cell analyses, performed at STI
- Influenza ELISA/HIT, performed at Pevion Biotech Ltd.

10.4 Statistical hypothesis

PEV 301 and PEV 302 are safe for inoculation into non-immune subjects. No serious adverse events attributable to either vaccine occur in the study population. One or both of the vaccines induce specific immune responses in at least 1/3 of the volunteers.

10.5 Sample size calculation

The sample size of this pilot study is determined by the requirement to determine safety of each formulation and dose. A sample size of 8 in each group constitute a reasonable sample size to estimate the frequency of AEs with an acceptable accuracy, allowing for dropouts. The study is not powered to ensure that differences in safety or immunogenicity between regimens will be statistically significant.

10.6 Statistical analysis

Demographic data of each study group will be tabulated.

Safety data:

Listings will be made of the safety data collected at each time point.

Descriptive statistics will be used to analyze adverse events (AEs) including intercurrent illnesses. The number of AEs and their severity will be reported using frequency tables. With frequently occurring (10 or more) event types, effects of dose and vaccine will be tested by

recording the presence/absence of the event in each patient and using logistic regression models to test for differences between groups.

Immunogenicity data:

Immunological data for each time point will be analyzed separately.

a) Descriptive statistics (minimum, maximum, median, geometric mean, arithmetic mean and quartiles) will be computed for each immunological measure and each time point, separately for each group.

b) For each volunteer, the ratio of the immunological measure to that assessed at baseline (during screening) will be computed. Descriptive statistics for these ratios (minimum, maximum, median, geometric mean, and quartiles) will be computed for each immunological measure and each time point, separately for each group.

c) Wilcoxon tests will be used to compare the immunological measures between doses, and between group E and groups C or D (as appropriate) for each immunological measure, each time point and each antigen.

10.7 Final analysis

The final analysis will be performed after evaluation of the data of the 3rd vaccination of groups E and F.

10.7.1 Safety evaluation

Safety of the injected study materials will be determined as the incidence of adverse events and the occurrence of significant clinical, hematological and biochemical abnormalities during the procedure and at the intervals indicated in the schedule of assessments. Listings will be made of the safety data collected at each time point.

No serious adverse events attributable to either vaccine are expected.

10.7.2 Immunogenicity evaluation

The criteria for the evaluation of immunogenicity of PEV 301 and PEV 302 will be the result of the following assays:

- ELISA for antibody titers against PEV 301 and PEV 302, performed by Pevion Biotech Ltd.
- Western Blotting and IFA for antibody titers crossreactive with *P. falciparum* parasites (blood stages and sporozoites, respectively), performed by STI

In addition, the following evaluations will be made:

- T-cell proliferation assays, performed at STI
- Parasite growth/invasion inhibition assays, performed at STI
- Isotyping, performed at STI
- ELISPOT and clonal T-cell analyses, performed at STI
- Influenza ELISA/HIT, performed at Pevion Biotech Ltd.

10.8 Interim analysis

An interim analysis will be performed after obtaining the results following the second injection of vaccine. Data will be collected in study week 13 (visit 9). If no immune response or a response considered being sufficient are seen in one of the study groups after 2 vaccinations, the study for this group will be terminated, i.e. no 3rd vaccination will be given.

10.9 Criteria for exclusion from analysis

Safety data analysis will be performed on all subjects who received at least one dose of study vaccine/virosomes, and for whom at least one set of safety follow-up data is available (i.e. safety population).

Subjects will be included in the final analysis of immunogenicity if they fulfill the following criteria (i.e. per protocol analysis):

- All inclusion/exclusion criteria were respected
- They received the vaccine they were randomized to receive
- They received a second dose of the vaccine
- Allowed intervals between injections were respected
- Allowed intervals between blood sampling schedules were respected
- No forbidden vaccine or concomitant medication was taken

11 Ethics and regulatory considerations

The study will be conducted according to Good Clinical Practice, the Declaration of Helsinki (Protocol Appendix A), Directive 2001/20/EC, Guideline for Good Clinical Practice CPMP/ICH/135/95, Protection of Human Subjects, Institutional Review Boards and local rules and regulations of the country.

11.1 Independent Ethics Committee

The protocol will be submitted to the Ethikkommission beider Basel (EKBB) for approval.

11.2 Informed consent

Written informed consent must be obtained from each subject participating in the screening visit. The method of explanation to the subject and the obtaining of their consent should comply with the ICH Guidelines, with local law and/or the ethical principles in the amended Declaration of Helsinki, whichever represents the greater protection for the individual. Pevion Biotech Ltd. / PMU/STI will provide PD Dr. Lukas Degen with subject consent forms to be signed by the subjects prior to starting their treatment as well as subject information sheets to be given to the subject prior to their consent.

For Information and Informed Consent Form see Appendix 2 and 3, respectively.

12 Administrative Matters

To comply with Good Clinical Practice important administrative obligations relating to investigator responsibilities, monitoring, archiving data, audits, confidentiality and publications must be fulfilled.

12.1 Protocol and Investigators Brochure (IB)

As it is essential that the study is carried out exactly in accordance with the terms of the protocol, PD Dr. Lukas Degen will familiarize himself fully with it to be able to conduct the study in the manner specified. Any amendments, revisions or modifications to the protocol must be in writing and agreed upon by both Parties prior to implementation.

Pevion Biotech Ltd. has supplied PD Dr. Lukas Degen with an Investigator’s Brochure (IB) which describes the vaccines being tested and its known adverse effects. PD Dr. Lukas

Degen must be familiar with this document before the study commences. Pevion Biotech Ltd. / PMU/STI will be happy to answer any questions PD Dr. Lukas Degen may have, either before commencing the studies or during their conduct.

12.2 Confidentiality

Either prior to or during the course of the study, Pevion Biotech Ltd. / PMU/STI or their representatives will provide PD Dr. Lukas Degen and persons delegated by him with confidential information, for example, but not limited to, the protocol and the IB. The information may not to be disclosed to anyone else without prior approval from Pevion Biotech Ltd. / PMU/STI in writing. This obligation of confidentiality shall survive the completion or early termination of the study.

12.3 Documentation and Material Supplies

All supplies provided to PD Dr. Lukas Degen for the purpose of carrying out the study are supplied only for the purpose of the study and must not be used for any other purpose whatsoever. PD Dr. Lukas Degen or (a) person(s) delegated by him is/are responsible for the security and accountability of all supplies. All such supplies, if not used during the course of the study and not forming a part of the documentation required to be retained by PD Dr. Lukas Degen, must be returned to Pevion Biotech Ltd. / PMU/STI at the conclusion of the study.

12.4 Financial compensation

All volunteers will receive financial compensation for their participation in the trial, which is intended to cover travel expenses and loss of other financial benefits they may lose by accepting to participate in the trial. Proposed amounts are CHF 800.-- at visit 12 and CHF 200.-- at visit 13. In case you decide to prematurely terminate the study participation you will receive a pro rata payment according to the duration of your participation. In case you have to withdraw from the study due to adverse events, you will receive the full payment.

12.5 Monitoring

The study will be monitored by the Pharmaceutical Medicine Unit (PMU) of the Swiss Tropical Institute (STI). Its representatives will be allowed access to all information resulting from this study and Pevion Biotech Ltd. will have an unrestricted right to use such information. The study monitor has to have access to laboratory test reports and other subject records needed to verify the entries on the CRF. The subjects' confidentiality will be respected as required by local law.

12.6 Source Documents and Case Report Forms (CRFs)

A CRF must be completed for each subject who enters the study and it is Dr. Lukas Degen’s responsibility to ensure that the CRFs are complete and accurate. Each case record form must be completed using black or blue ball point pens. Any mistakes must be crossed through with a single line, not obliterated or corrected with liquid paper (e.g. Tipp-Ex®), the correction added and signed and dated by the PD Dr. Lukas Degen.

Independent case histories for each subject and source documents must be maintained to support the data entered on the CRF and Dr. Lukas Degen commits to make all subject medical records and other source documents, which are pertinent to the Study available to Pevion Biotech Ltd. / PMU/STI and/or health authority representatives upon request. All subject records must be kept in a secure place for fifteen (15) years.

12.7 Quality assurance audit

This study may be audited by Pevion Biotech Ltd. / PMU-STI or its designee to document the authenticity of recorded data and protocol adherence. Subjects participating in the study should be informed that their records might be reviewed for this purpose, and also by government health authorities. The subjects' confidentiality will be respected as required by local law.

12.8 Compensation for medicine-induced injury

Pevion Biotech Ltd. assumes liability for and will indemnify all injuries that occur to trial subjects or subjects whenever a causal relationship can be established between the event and the clinical trial procedure or the trial substance under study if the following can be demonstrated:

a. The event resulted from a trial substance, provided that the substance was administered according to the approved PEVION trial protocol PEV 001.

b. The event arose in association with the use of comparative substances used legitimately as part of the trial protocol.

c. The event occurred as a consequence of diagnostic procedures performed according to the trial protocol.

d. The event resulted from therapeutic or diagnostic measures legitimately required as a consequence of unexpected events caused by the trial substance, by comparative medication, or by diagnostic procedures called for by the trial protocol.

Pevion Biotech Ltd is not liable for events that occur solely as a consequence of the underlying illness of the trial subject or subject, or for events resulting from diagnostic or therapeutic measures not specifically required by the trial protocol, or for events resulting from negligence (including failure to act according to accepted medical practice, or to comply strictly with the protocol or the terms of this Agreement) of PD Dr. Lukas Degen or any other involved and/or related clinical staff and facilities.

This indemnity provided by Pevion Biotech Ltd shall further apply as follows:

a. Pevion Biotech Ltd. is to be informed as soon as possible of any complaint, action or suit of proceeding giving rise to the right of indemnification, and PD Dr. Lukas Degen agrees to co-operate fully with Pevion Biotech Ltd in the defense or disposition of all such cases.

b. Pevion Biotech Ltd will be permitted, at its costs and discretion, to handle and control the defense or disposition of all such cases.

c. No case will be settled without the prior written consent of Pevion Biotech Ltd.

12.9 Early termination

It is Pevion's intention that this study is carried out to its conclusion, but PD Dr. Lukas Degen must be aware that for a number of reasons, the study may need to be stopped prior to their conclusion. Pevion Biotech Ltd., therefore, reserves the right to terminate this Agreement:

a. immediately upon a substantial breach of the terms either of the Agreement or of the conduct of the protocol;

b. in the event of irregularities in the method by which the study is carried out and although capable of being rectified, are not rectified within thirty (30) days of notice from Pevion Biotech Ltd. / PMU/STI requiring this.

c. immediately, if this is necessary in the interests of health and safety of the study subjects, or as a result of an order of any government authority or court of law.

In the event of early termination, PD Dr. Lukas Degen will cease use of the investigational drug immediately. All CRFs outstanding must be completed and returned to Pevion Biotech Ltd. together with completed drug inventory, records and remaining trial material.

12.10 Publication of data and protection of trade secrets

In accord with standard editorial and ethical practice, Pevion Biotech Ltd. will support publication of multi-center trials only in their entirety and not as individual center data except for data on substudies.

PD Dr. Lukas Degen agrees to submit all manuscripts or abstracts to Pevion Biotech Ltd. prior to submission. This allows Pevion Biotech Ltd. to protect proprietary information and to provide comments based on information from other studies that may not yet be available to the authors of the manuscript. PD Dr. Lukas Degen will collaborate with the study statistician for preparation of trial data analyses intended to be used in the publication(s) of the study.

Any formal publication of the study in which input of Pevion Biotech Ltd. personnel exceeded that of conventional monitoring will be considered as a joint publication by PD Dr. Lukas Degen and Pevion Biotech Ltd.

Moreover, the following points need to be considered:

a. Without PD Dr. Lukas Degen's prior written consent, Pevion Biotech Ltd may not make reference, either directly or indirectly, in a commercial publication, to PD Dr. Lukas Degen's name or institution, or any of its employees in which PD Dr. Lukas Degen performed the present trials, connected with the research and its results.

b. Pevion Biotech Ltd. may not use PD Dr. Lukas Degen's name or the name of the Kantonsspital Basel or its employees connected to the research or to the institution in which PD Dr. Lukas Degen performed the present trial in its commercial publications as recommences of quality and/or of the finished product and/or of the drug and the efficacy of its use.

c. Should Pevion Biotech Ltd. decide to publish the research results, it must publish them in their entirety and must not quote anything out of context.

Nothing in the aforementioned limitations in clauses a.-c. will prevent Pevion Biotech Ltd. from quoting from articles, provided that the scientific source of data (scientific conventions, scientific newspapers) is mentioned.

12.11 Intellectual property rights

Data resulting from this study shall be the sole property of Pevion Biotech Ltd. Should any inventions/improvements result from this study, Pevion Biotech Ltd. shall be entitled to file in its own name relevant patent applications, and the said inventions and improvements will become and remain the sole property of Pevion Biotech Ltd. PD Dr. Lukas Degen agrees to provide Pevion Biotech Ltd. with all requested assistance necessary for obtaining any

patents, including execution of legal documents. It is understood that any publication is withheld until patent application is filed.

12.12 Entry violations

All subjects, including those who enter the study but are found to violate the entry criteria must be followed to the end of the study.

13 References

1. Bisang, C, Jiang L, Freund, E, Emery, F, Bauch, C, Matile, H, Pluschke, G, Robinson, J. A. Synthesis, conformational properties, and immunogenicity of a cyclic template-bound peptide mimetic containing an NPNA motif from the circumsporozoite protein of *Plasmodium falciparum.* J Am Chem Soc 1998;120:7439 – 7449
2. Pöltl-Frank, F, Zurbriggen, R, Helg, A, Stuart, F, Robinson, J, Glück, R, Pluschke, G Use of reconstituted influenza virus virosomes as an immunopotentiating delivery system for a peptide-based vaccine. Clin Exp Immunol 1999;117:496-503
3. Pfeiffer, B, Moreno, R, Moehle, K, Zurbriggen, R, Glück, R, Pluschke, G, and Robinson, J A, Application of protein epitope mimetics in vaccine design. Chimia 2001;55:334 - 339
4. Moreno, R, Jiang, L, Moehle, K, Zurbriggen, R, Glück, R, Robinson, JA and Pluschke, G Exploiting conformationally constrained peptidomimetics and an efficient human-compatible delivery system in synthetic vaccine design. Chem Bio Chem 2001;2:838 - 843
5. Pfeiffer B, Peduzzi E, Moehle K, Zurbriggen R, Glück R, Pluschke G and Robinson JA A virosome-mimotope (Viro-Tope) approach to synthetic vaccine design and optimization: synthesis, conformation and immune recognition of a new potential malaria vaccine candidate. Angewandte Chemie 2003;42:2368 – 2371
6. Mueller MS, Renard A, Boato F, Vogel D, Naegeli M, Zurbriggen R, Robinson JA, and Pluschke G Induction of Parasite Growth Inhibitory Antibodies by a Virosomal Formulation of a Peptidomimetic of Loop-I from Domain III of *Plasmodium falciparum.* Infect Immun 2003;71:4758-4779

Appendix 1: Helsinki Declaration

World Medical Association Declaration of Helsinki

Recommendations guiding physicians in biomedical research involving human subjects.

Adopted by the 18th World Medical Assembly, Helsinki, Finland, June 1964, amended by the 29th World Medical Assembly, Tokyo, Japan, October 1975, the 35th World Medical Assembly, Venice, Italy, October 1983, the 41st World Medical Assembly, Hong Kong, September 1989, and the 48th General Assembly, Somerset West, Republic of South Africa, October 1996.

Introduction

It is the mission of the physician to safeguard the health of the people. His or her knowledge and conscience are dedicated to the fulfillment of this mission.

The Declaration of Geneva of the World Medical Association binds the physician with the words, "The Health of my patient will be my first consideration," and the International Code of Medical Ethics declares that, "A physician shall act only in the patient's interest when providing medical care which might have the effect of weakening the physical and mental condition of the patient.

The purpose of biomedical research involving human subjects must be to improve diagnostic, therapeutic and prophylactic procedures and the understanding of the etiology and pathogenesis of disease.

In current medical practice most diagnostic, therapeutic or prophylactic procedures involve hazards. This applies especially to biomedical research.

Medical progress is based on research, which ultimately must rest in part on experimentation involving human subjects.

In the field of biomedical research a fundamental distinction must be recognized between medical research in which the aim is essentially diagnostic or therapeutic for a patient, and medical research, the essential object of which is purely scientific and without implying direct diagnostic or therapeutic value to the person subjected to the research.

Special caution must be exercised in the conduct of research, which may affect the environment, and the welfare of animals used for research must be respected.

Because it is essential that the results of laboratory experiments be applied to human beings to further scientific knowledge and to help suffering humanity, the World Medical Association has prepared the following recommendations as a guide to every physician in biomedical research involving human subjects. They should be kept under review in the future. It must be stressed that the standards as drafted are only a guide to physicians all over the world. Physicians are not relieved from criminal, civil and ethical responsibilities under the laws of their own countries.

I. Basic Principles

- Biomedical research involving human subjects must conform to generally accepted scientific principles and should be based on adequately performed laboratory and animal experimentation and on a thorough knowledge of the scientific literature.
- The design and performance of each experimental procedure involving human subjects should be clearly formulated in an experimental protocol which should be transmitted for consideration, comment and guidance to a specially appointed committee independent of the investigator and the sponsor provided that this independent committee is in conformity with the laws and regulations of the country in which the research experiment is performed.

- Biomedical research involving human subjects should be conducted only by scientifically qualified persons and under the supervision of a clinically competent medical person. The responsibility for the human subject must always rest with a medically qualified person and never rest on the subject of the research, even though the subject has given his or her consent.
- Biomedical research involving human subjects cannot legitimately be carried out unless the importance of the objective is in proportion to the inherent risk to the subject.
- Every biomedical research project involving human subjects should be preceded by careful assessment of predictable risks in comparison with foreseeable benefits to the subject or to others. Concern for the interests of the subject must always prevail over the interests of science and society.
- The right of the research subject to safeguard his or her integrity must always be respected. Every precaution should be taken to respect the privacy of the subject and to minimize the impact of the study on the subject's physical and mental integrity and on the personality of the subject.
- Physicians should abstain from engaging in research projects involving human subjects unless they are satisfied that the hazards involved are believed to be predictable. Physicians should cease any investigation if the hazards are found to outweigh the potential benefits.
- In publication of the results of his or her research, the physician is obliged to preserve the accuracy of the results. Reports of experimentation not in accordance with the principles laid down in this Declaration should not be accepted for publication.
- In any research on human beings, each potential subject must be adequately informed of the aims, methods, anticipated benefits and potential hazards of the study and the discomfort it may entail. He or she should be informed that he or she is at liberty to abstain from participation in the study and that he or she is free to withdraw visor her consent to participation at any time. The physician should then obtain the subject's freely given informed consent, preferably in writing.
- When obtaining informed consent for the research project the physician should be particularly cautious if the subject is in a dependent relationship to him or her or may consent under duress. In that case the informed consent should be obtained by a physician who is not engaged in the investigation and who is completely independent of this official relationship.
- In case of legal incompetence, informed consent should be obtained from the legal guardian in accordance with national legislation. Where physical or mental incapacity makes it impossible to obtain informed consent, or when the subject is a minor, permission from the responsible relative replaces that of the subject in accordance with national legislation.

  Whenever the minor child is in fact able to give a consent, the minor's consent must be obtained in addition to the consent of the minor's legal guardian.
- The research protocol should always contain a statement of the ethical considerations involved and should indicate that the principles enunciated in the present Declaration are complied with.

II. Medical Research Combined with Professional Care (Clinical Research)

- In the treatment of the sick person, the physician must be free to use a new diagnostic and therapeutic measure, if in his or her judgment it offers hope of saving life, re-establishing health or alleviating suffering.
- The potential benefits, hazards and discomfort of a new method should be weighed against the advantages of the best current diagnostic and therapeutic methods.
- In any medical study, every patient- including those of a control group, if any- should be assured of the best proven diagnostic and therapeutic method. This does not exclude the use of inert placebo in studies where no proven diagnostic or therapeutic method exists.

- The refusal of the patient to participate in a study must never interfere with the physician-patient relationship.
- If the physician considers it essential not to obtain informed consent, the specific reasons for this proposal should be stated in the experimental protocol for transmission to the independent committee (1, 2).
- The physician can combine medical research with professional care, the objective being the acquisition of new medical knowledge, only to the extent that medical research is justified by its potential diagnostic or therapeutic value for the patient.

III. Non-Therapeutic Biomedical Research Involving Human Subjects (Non-Clinical Biomedical Research)

- In the purely scientific application of medical research carried out on a human being, it is the duty of the physician to remain the protector of the life and health of that person on whom biomedical research is being carried out.
- The subjects should be volunteers- either healthy persons or patients for whom the experimental design is not related to the patient's illness.
- The investigator or the investigating team should discontinue the research if in his/her or their judgment it may, if continued, be harmful to the individual.
- In research on man, the interest of science and society should never take precedence over considerations related to the well-being of the subject.

Appendix 2: Subject Information Sheet

Kantonsspital Basel
Clinical Research Center

Information über die klinische Studie mit Komponenten eines Malaria-Impfstoffes

Protokoll-Nummer	PEV 001
Studien-Titel	A phase I open randomized single blind study of two virosome formulated anti-malaria vaccine components (PEV 301 and PEV 302) administered alone and in combination to healthy adult volunteers
Verantwortlicher Prüfarzt	PD Dr. Lukas Degen<br>Clinical Research Center<br>Kantonsspital Basel<br>Tel. 061 265 51 74<br>e-mail: lukas.degen@unibas.ch
Auftraggeber	Pevion Biotech AG<br>Rehagstrasse 79<br>3018 Bern<br>Tel. 031 980 64 17

Name des Teilnehmenden: ______________________________
Randomisierungs-Nr.: ______________________________
Adresse des Teilnehmenden: ______________________________
Telefon-Nummer: ______________________________

Sehr geehrte Damen und Herren, liebe Probandin, lieber Proband

Sie haben sich bereit erklärt, an den Vorabklärungen für die Teilnahme an einer klinischen Studie teilzunehmen. Bevor Sie sich definitiv entscheiden, lesen Sie bitte diese Information ausführlich durch und fragen Sie bei Unklarheiten den Prüfarzt.

1. **Ziel der Studie:** Mit dieser Studie wird wissenschaftlich geprüft, ob die vom Schweizerischen Tropeninstitut in Vorversuchen als immunogen identifizierten Malaria-Antigene auch im Menschen eine Immunantwort hervorrufen. Als Transportsystem werden Virosomen gewählt, wie sie schon seit Jahren für die Grippe-Impfung verwendet werden. Eine Infektion mit Malariaerregern findet nicht statt.

2. **Auswahl und Zuteilung der Studienteilnehmer zu den Gruppen:** Falls Sie alle Einschluss- und Ausschlusskriterien erfüllen. Mit einer vom Statistiker hergestellten Zufallsliste werden Sie einer der Gruppen zugeteilt, d.h. Sie werden entweder 3 x entweder mit 10 µg PEV 301 oder mit 50 µg PEV 301 oder mit PEV 302 (10 µg oder 50 µg oder mit der Kombination von beiden (50 µg PEV 301 plus 50 µg PEV 302) geimpft oder sie bekommen nur die Virosomen allein. Total werden 46 freiwillige Probanden eingeschlossen (8 pro aktive Gruppe, 6 bei der Virosomen-Gruppe), wenn möglich gleichviele Frauen wie Männer).

3. **Durchführung der Studie**: Die Studie beginnt für die 10 µg-Gruppen mit einer Voruntersuchung anfangs November. Teilnehmende, die alle Ein- und Ausschlusskriterien erfüllen und bereit sind, für alle Termine ans Clinical Research Center des Kantonsspitals Basel zu kommen, werden in die Studie aufgenommen.
   Die 3 Impfungen (3x derselbe Impfstoff/Gruppe) werden durchgeführt am Tag 1, 60 und 180 der Studie. Aus Sicherheitsgründen werden Sie während mind. 30 Minuten nach der Impfung im Kantonsspital beobachtet, nachher erhalten Sie einen kleinen Fragebogen, in den Sie eintragen können, wie Sie die Impfungen vertragen haben.

Ca. 48 Stunden nach der Impfung werden Sie aufgeboten zum Berichten allfälliger unerwünschter Ereignisse in Zusammenhang mit der Impfung. Sollte an der Impfstelle eine Reaktion sichtbar sein, wird ein Foto der Impfstelle gemacht.
An den Tagen 8 und 22 nach jeder Impfung wird Blut entnommen zur Erfassung allfälliger Abweichungen von den Grundwerten, die während der Voruntersuchung bestimmt wurden.
Zur Bestimmung der Immun-Antwort wird Blut entnommen während der Voruntersuchung, vor der Impfung am Tag 60 und 180 sowie während der Kontroll-Untersuchungen an den Tagen 22 nach jeder Impfung.

4. **Schwangerschaftsverhütung:** Zur Abklärung der Einschlusskriterien wird bei Frauen ein Schwangerschaftstest durchgeführt, Sie dürfen nur an der Studie teilnehmen, wenn dieser Test negativ ist. Zudem sind Sie verpflichtet, während der ganzen Dauer der Studie eine wirksame Schwangerschaftsverhütungsmethode zu benutzen.

5. **Nutzen für zukünftige Patientinnen und Patienten:** Diese Studie ist Teil der Entwicklung eines neuen Malaria-Impfstoffes. Ihre Teilnahme an der Studie bringt wichtige neue Kenntnisse zur Wirkung des Impfstoffes, der später einmal in Malaria-Gebieten eingesetzt werden könnte. Sie selber haben jedoch keinen prophylaktischen oder therapeutischen Nutzen aus dieser Studie.

6. **Risiken einer Teilnahme:** Die Impfstoffe werden in Ihren Oberarm gespritzt, es ist möglich, dass die Einstichstelle schmerzt oder dass sogar Rötungen oder Schwellungen auftreten. Bedingt durch die Blutentnahmen können lokale Schmerzen auftreten. Systemische Reaktionen (wie schwaches Fieber, grippeähnliche Symptome und allergische Reaktionen) können bei jeder Impfung nicht ganz ausgeschlossen werden.

7. **Freiwillige Teilnahme**: Ihre Teilnahme an dieser klinischen Studie erfolgt freiwillig, nach ausführlicher Information und Beantwortung aller Ihrer Fragen. Sie können sich jederzeit, auch ohne Angabe von Gründen, von der Studie zurückziehen. In diesem Falle sind Sie gebeten, mit dem CRC einen Termin für die Austrittsuntersuchung zu vereinbaren.

8. **Unfreiwilliger Studienabbruch:** Der Prüfarzt kann Sie im Interesse Ihrer Gesundheit aus dieser Studie ausschliessen. Folgende Gründe können dazu führen:
Nebenwirkungen, Schwangerschaft bei Frauen, Reisen in ein Malariagebiet.

9. **Vertraulichkeit der Daten**: Wenn Sie einverstanden sind, an dieser Studie teilzunehmen und die Einverständniserklärung unterschreiben, willigen Sie ein, dass Beauftragte des Auftraggebers, der Gesundheitsbehörden und der Ethikkommission, die diese Studie genehmigt hat, sowie im Versicherungsfalle ein Vertreter der Versicherungsgesellschaft, Zugang zu Ihren medizinischen Aufzeichnungen erhalten. Ihre medizinischen Daten sind notwendiger Bestandteil der Studie und werden einer elektronischen Datenverarbeitung unterzogen. Sie werden nur an den Auftraggeber oder wenn nötig an die entsprechenden Gesundheitsbehörden weitergegeben, unter Bedingungen, die die Vertraulichkeit garantieren.

10. **Versicherung:** Die Firma Pevion Biotech AG ersetzt Ihnen Schäden, die Sie gegebenenfalls im Rahmen des klinischen Versuchs erleiden. Zu diesem Zweck hat die Firma Pevion Biotech AG zu Ihren Gunsten eine Versicherung bei der Versicherung Gerling Konzern abgeschlossen. Stellen Sie während oder nach dem klinischen Versuch gesundheitliche Probleme oder andere Schäden fest, so wenden Sie sich bitte an den verantwortlichen Arzt (PD Dr. Lukas Degen). Ihr Prüfarzt weiss über die geltende Gesetzgebung Bescheid, verfügt über die entsprechenden schriftlichen Unterlagen und wird für Sie die notwendigen Schritte einleiten.

11. **Vergütung:** Für Ihre Bereitschaft, an dieser wichtigen Studie teilzunehmen, möchten wir Ihnen vielmals danken. Für Ihren Zeitaufwand und die Blutentnahmen erhalten Sie eine Vergütung von CHF 1000.--. Falls Ihre Teilnahme an der Studie aus gesundheitlichen Gründen, z.B. bei Nebenwirkungen, abgebrochen werden muss, wird Ihnen der ganze Betrag ausbezahlt. Falls Sie die Studie aus eigener Entscheidung vor dem Studienende verlassen, wird Ihnen ein pro rata Betrag, basierend auf den Studientagen, an denen Sie teilgenommen haben, ausbezahlt.

12. **Kontaktperson:** Bei Unklarheiten, Notfällen, unerwarteten oder unerwünschten Ereignissen, die während der Studie oder bis zu 3 Monaten nach deren Abschluss auftreten, können Sie sich jederzeit an die untenstehende Kontaktperson wenden:

Dr. med. Lukas Degen
Clinical Research Center, Kantonsspital Basel
Tel 061 265 51 74

Ich habe die mir abgegebene schriftliche Information über die Studie gelesen und verstanden. Meine Fragen im Zusammenhang mit der Teilnahme an dieser Studie wurden mir zufrieden stellend beantwortet. Ich kann diese Information behalten.

Appendix 3: Informed Consent Agreement

Kantonsspital Basel
Clinical Research Centre

Schriftliche Einverständniserklärung zur Teilnahme an der klinischen Studie mit Komponenten eines Malaria-Impfstoffes

Bitte lesen Sie dieses Formular sorgfältig durch und fragen Sie, falls Sie etwas nicht verstehen oder etwas wissen möchten.

- Ich wurde vom unterzeichnenden Arzt des Clinical Research Center des Kantonsspitals Basel, PD Dr. L. Degen, mündlich und schriftlich informiert über die Ziele und den Ablauf der Studie mit virosome formulierten Malaria Mimetics. Ich weiss, dass kein Risiko besteht, an einer Malaria zu erkranken, da keine Infektion geplant ist. Ich wurde ausserdem bezüglich Risiken, meinem persönlichen Nutzen und der freiwilligen Teilnahme an der Studie informiert.

- Ich weiss, dass ich nur in die Studie aufgenommen werde, wenn ich alle Einschlusskriterien erfülle und keines der Ausschlusskriterien zutrifft.

- Ich habe die schriftliche Information über die Studie gelesen und verstanden. Meine Fragen im Zusammenhang mit der Teilnahme an dieser Studie wurden mir zufrieden stellend beantwortet. Ich hatte genügend Zeit, um meine Entscheidung zu treffen.

- Ich bin darüber informiert, dass eine Versicherung Gesundheitsschäden deckt, falls solche im Rahmen und im Zusammenhang mit der Studie auftreten.

- Ich nehme freiwillig an dieser Studie teil, d.h. ich kann jederzeit und ohne Angabe von Gründen meine Zustimmung zur Teilnahme widerrufen. In diesem Fall werde ich zu meiner Sicherheit abschliessend medizinisch untersucht.

- Ich bin einverstanden, dass die zuständigen Fachleute des Studienauftraggebers, der Behörden und der Ethikkommissionen zu Prüf- und Kontrollzwecken in meine Originaldaten Einsicht nehmen dürfen, jedoch unter strikter Einhaltung der Vertraulichkeit.

- Ich kann die schriftliche Probandeninformation behalten und ich erhalte eine Kopie dieser Einverständniserklärung.

Unterschrift des Teilnehmers	Name in Blockschrift	Datum der Unterschrift

Ich, der unten Unterzeichnende, bestätige hiermit, dass ich alle relevanten Informationen zu dieser klinischen Studie dem oben genannten Teilnehmer weitergegeben habe. Ich werde ihm eine Kopie dieses unterzeichneten Dokumentes mit der Einwilligung zur Studienteilnahme zukommen lassen.

Unterschrift des Prüfarztes oder seines Vertreters	Name in Blockschrift	Datum der Unterschrift

Appendix 4: Common Terminology Criteria

The Common Terminology Criteria for Adverse Events v3.0 (CTCAE) will be used for grading and reporting of Adverse Events during the trial.

The reference document is provided as a separate document to the CRC, Kantonsspital Basel.
